# Whole-Genome Resequencing of Near-Isogenic Lines Reveals a Genomic Region Associated with High *Trans*-Lycopene Contents in Watermelon

**DOI:** 10.3390/plants11010008

**Published:** 2021-12-21

**Authors:** Siyoung Lee, Girim Park, Yunseo Choi, Seoyeon Park, Hoytaek Kim, Oakjin Lee, Taebok Kim, Younghoon Park

**Affiliations:** 1 Department of Horticultural Bioscience, Pusan National University, Miryang 50463, Korea; warain74123@naver.com (S.L.); rlfla007@gmail.com (G.P.); chldudal1000@naver.com (Y.C.); pal3437@naver.com (S.P.); 2 Department of Horticulture, Sunchon National University, Sunchon 57922, Korea; htkim@scnu.ac.kr; 3 National Institute of Horticultural and Herbal Science, Rural Development Administration, Wanju 55365, Korea; ojlee6524@korea.kr (O.L.); biok@korea.kr (T.K.)

**Keywords:** *trans*-lycopene, red-fleshed watermelon, whole-genome resequencing, cleaved amplified polymorphic sequence marker

## Abstract

*Trans*-lycopene is a functional phytochemical abundant in red-fleshed watermelons, and its contents vary among cultivars. In this study, the genetic basis of high *trans*-lycopene contents in scarlet red flesh was evaluated. Three near-isogenic lines (NILs) with high *trans*-lycopene contents were derived from the scarlet red-fleshed donor parent DRD and three coral red-fleshed (low *trans*-lycopene contents) recurrent parents. The lycopene contents of DRD (589.4 ± 71.8 µg/g) were two times higher than that of the recurrent parents, and values for NILs were intermediate between those of the parents. Coral red-fleshed lines and F1 cultivars showed low *trans*-lycopene contents (135.7 ± 18.0 µg/g to 213.7 ± 39.5 µg/g). Whole-genome resequencing of two NILs and their parents and an analysis of genome-wide single-nucleotide polymorphisms revealed three common introgressed regions (CIRs) on chromosomes 6, 9, and 10. Twenty-eight gene-based cleaved amplified polymorphic sequence (CAPS) markers were developed from the CIRs. The CAPS markers derived from CIR6 on chromosome 6, spanning approximately 1 Mb, were associated (*R*^2^ = 0.45–0.72) with the *trans*-lycopene contents, particularly CIR6-M1 and CIR6-M4. Our results imply that CIR6 is a major genomic region associated with variation in the *trans*-lycopene contents in red-fleshed watermelon, and CIR6-M1 and CIR6-M4 may be useful for marker-assisted selection.

## 1. Introduction

Watermelon (*Citrullus lanatus* var. *lanatus*; 2n = 2x = 22) is a desert plant native to Africa and a widely cultivated vegetable fruit crop in temperate climates. Watermelon production in 2019 was more than 32 tons per hectare, and more than 5790 kilotons were produced worldwide, making it an economically important crop (FAOSTAT, 2020). The flesh of watermelon is rich in sugars, such as glucose, fructose, and sucrose, and various nutrients, including carotenoid components, vitamins (A, B, C, E), and minerals (K, Mg, Ca, Fe), along with a large amount of water. In addition to hydration, it has great nutritional value [1]. In particular, carotenoids are abundant phytochemicals in watermelon fruit flesh, and lycopene is a major carotenoid-based compound along with beta-carotene and lutein [2]. Lycopene has anti-oxidative and anti-inflammatory effects as well as chemopreventive biological activity [3].

The flesh color of watermelon is broadly classified into red, yellow, orange, and white (UPOV, 2012). Differences in flesh color correspond to the differences in the composition of carotenoids and the relative amount of each carotenoid component [4,5]. In red flesh, *trans*-lycopene accounts for approximately 91% of the total carotenoids [6,7,8]. Red flesh in watermelons is typically classified as coral red (pink) and scarlet red depending on the intensity of the red flesh color [9]. In the case of orange-colored flesh, the main carotenoid component is pro-lycopene or beta-carotene, accounting for approximately 61–71% of the total carotenoids [4,5,10]. For yellow flesh, all-*trans*-violaxanthin, 9-cis-violaxanthin, and luteoxanthin are the main carotenoids; however, the total carotenoid contents are only 1–2% of that of red or orange watermelon [5]. Carotenoids have not been detected in white-fleshed watermelon [4].

Variation in the flesh color and carotenoid components is directly regulated by variation in genes encoding the enzymes involved in the carotenoid biosynthetic pathway. The accumulation of pro-lycopene in orange flesh is related to mutations in the carotenoid isomerase (*CRISTO*) gene on chromosome 4 [10]. Genes that regulate beta-carotene accumulation in orange-colored flesh have not yet been identified; however, a major quantitative trait locus (QTL) has been detected on chromosome 1 [11]. Loss-of-function mutations in the lycopene β-cyclase (*LCYB*) gene on chromosome 2 are responsible for the accumulation of *trans*-lycopene in red flesh [12], while wild-type *LCYB* is detected in yellow flesh. However, to the best of our knowledge, the genetic mechanisms or genes that regulate *trans*-lycopene levels in scarlet- and coral red-fleshed watermelons have not been reported.

Near-isogenic lines (NILs) are often used in identifying trait-related genes using genetic association studies in various crops, such as rice, soybean, and wheat [13,14,15]. NILs are developed by repeated backcrosses between a donor parent and a recurrent parent, followed by self-pollination to fix the genotype. Consequently, NILs show the targeted trait introduced from the donor parent, and the genomic background is recovered from the recurrent parent [16]. Therefore, when comparing NILs with the same target trait, QTL analyses, gene cloning, and development of trait-related molecular markers can be conducted based on the theory that the gene underlying the target trait is likely to exist in the chromosomal region where genetic variation is detected [17]. For example, molecular markers associated with powdery mildew resistance have been developed for watermelon using a NIL population [18]. In addition, whole-genome resequencing (WGRS) technology can efficiently decipher the genome sequences of NILs for more rapid and accurate determination of introgressed regions in NILs compared with conventional molecular marker-based methods [19,20].

Therefore, in this study, genetic data were obtained to clarify the molecular mechanism underlying variation in *trans*-lycopene contents between coral red- and scarlet red-fleshed watermelon varieties. In particular, an introgressed region associated with high *trans*-lycopene contents was identified based on full-length genome analysis of NILs. A set of molecular markers within this region was developed for marker-assisted selection for high lycopene (HL) contents.

## 2. Results

### 2.1. Lycopene Contents Analysis

**Figure 1:**
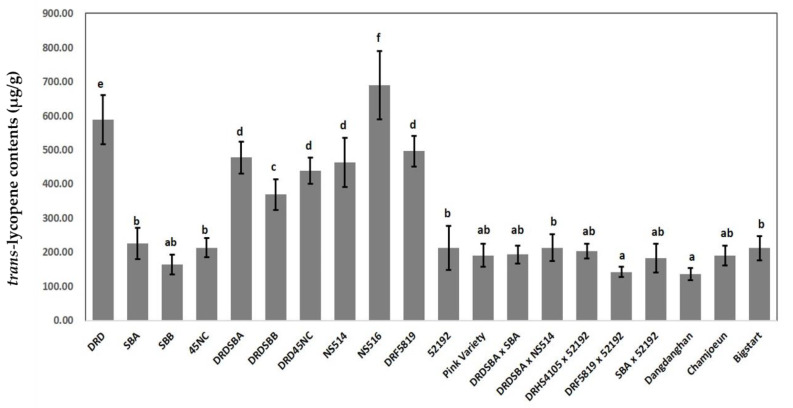
Variations in the *trans*-lycopene contents (µg/g) of fruit flesh among 20 watermelon cultivars. Differences between cultivars were determined at the 5% significance level using one-way ANOVA and Duncan’s multiple range tests.

Lycopene contents were analyzed in five watermelon fruits for each of 20 cultivars (Table 1, Figure 1, Table S1). The lycopene contents of DRD, the donor parent for the three NILs with HL contents, was 589.4 ± 71.8 µg/g, whereas the lycopene contents of SBA, SBB, and 45NC, the recurrent parents with low lycopene (LL) contents, were 225.83 ± 45.0, 164.0 ± 28.5, and 213.4 ± 28.2 µg/g, respectively. The lycopene contents of NILs (DRDSBA, DRDSBB, and DRD45NC; see Figure 1) were approximately intermediate between those of the HL and LL parents. Coral red-fleshed cultivars 52192 and Pink Variety showed LL contents of 213.1 ± 63.6 and 191.0 ± 34.8 µg/g, respectively, and all eight F_1_ hybrids also showed lycopene contents of between 135.7 ± 18.0 and 213.7 ± 39.5 µg/g. Overall, the lycopene contents of scarlet red-fleshed cultivars, NILs, and coral red-fleshed cultivars differed significantly (Table 1).

### 2.2. WGRS

The HL donor DRD, LL recurrent parents SBA and 45NC, and NILs (DRDSBA and DRD45NC) were subjected to next-generation sequencing (NGS) (Table 2). The number of reads generated for each DNA sample, average read length, total length of generated reads, and genome coverage are summarized in Table 2. The number of trimmed reads ranged from 57,130,281 (DRD45NC) (approximately 80% of the number of raw reads) to 78,305,938 (DRDSBA) (approximately 77% of the number of raw reads), and the average read length ranged from 88.58 to 92.80 bp. The total read length was at least 5,090,776,571 bp and up to 7,244,678,852 bp. Genome coverage was obtained by dividing the size of the reference genome sequence (97103 genome assembly version 2) by the total read length; the minimum value was 24.44× (DRD45NC), and the maximum was 33.40× (DRDSBA).

### 2.3. Detection of SNPs and Introgressed Regions in NILs

A total of 213,920 SNPs and 11,695 InDels were detected in the comparison between DRD and SBA. Among these variants, 12,750 SNPs and 498 InDels alleles for DRD were detected in DRDSBA. In addition, among the 216,329 SNPs and 11,606 InDels detected between DRD and 45NC, 13,864 SNPs and 665 InDels alleles for DRD were detected in DRD45NC.

**Figure 2:**
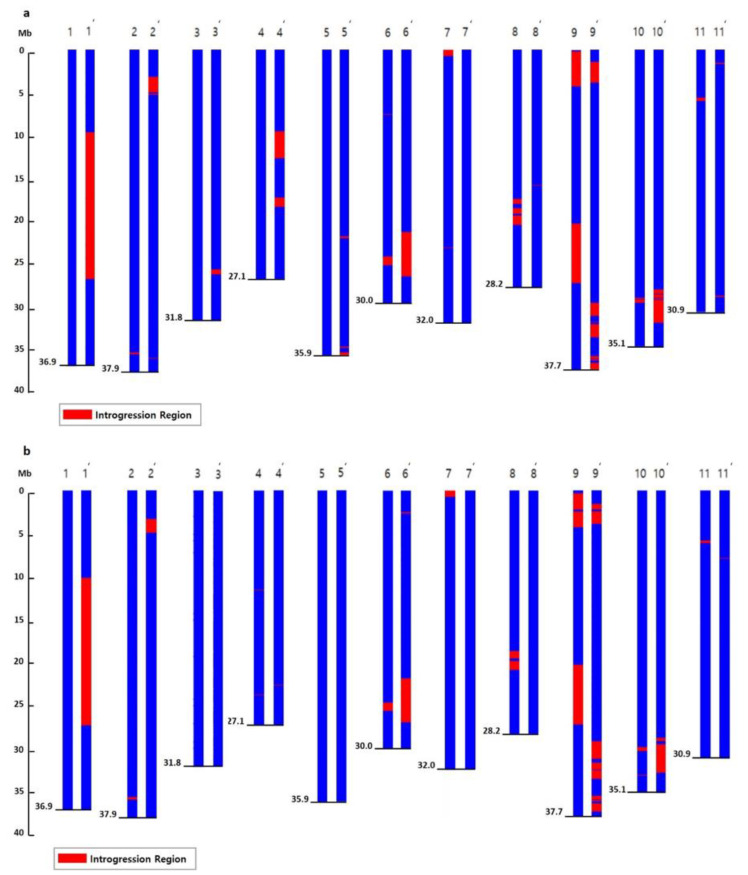
Schematic representation of 11 the chromosomes (blue) in DRDSBA (left) and DRD45NC (right) showing introgressed genomic regions (red) derived from DRD. The introgressed regions were revealed by an analysis of single-nucleotide polymorphisms (SNPs) (**a**) and insertions/deletions (InDels) (**b**) based on the whole-genome resequencing.

The number and genomic distribution of these SNPs on each chromosome are shown in Figure S1. The introgressed regions of the donor DRD chromosome in the two NILs were estimated based on the distribution of DRD-specific SNPs and InDels in the NIL genomes. In SNPs, for DRDSBA, the introgressed regions were detected in Chr. 6 (7–8 Mb, 24–26 Mb), Chr. 7 (0–1 Mb), Chr. 8 (18–21 Mb), Chr. 9 (about 0–5 Mb, 20–27 Mb), and Chr. 10 (27–28 Mb, 29–30 Mb). DRD45NC showed the introgressed regions in Chr. 1 (10–27 Mb), Chr. 2 (3–6 Mb), Chr. 4 (11–12 Mb, 18–19 Mb), Chr. 6 (21–27 Mb), Chr. 9 (1–4 Mb, 27–28 Mb, 29–34 Mb, 35–38 Mb), and Chr. 10 (28–33 Mb) (Figure 2, Figure S2). In InDels, for DRDSBA, the introgressed regions were detected in Chr.2 (35–36 Mb, 37–38 Mb), Chr.6 (7–8 Mb, 24–26 Mb), Chr.7 (0–1 Mb, 4–5 Mb, 22–24 Mb), Chr.8 (4–5 Mb, 18–21 Mb), Chr.9 (0–5 Mb, 20–27 Mb), Chr.10 (2–3 Mb, 27–28 Mb, 29–30 Mb). DRD45NC showed the introgressed regions in Chr.1 (2–3 Mb, 10–15 Mb, 16–19 Mb, 20–27 Mb), Chr.2 (3–5 Mb), Chr.3 (3–5 Mb), Chr.6 (2–3 Mb, 21–27 Mb), Chr.9 (1–4 Mb, 26–27 Mb, 29–34 Mb, 35–38 Mb), Chr.10 (12–13 Mb, 28–33 Mb).

### 2.4. Detection of Common Introgressed Regions (CIRs) and SNPs

**Figure 3:**
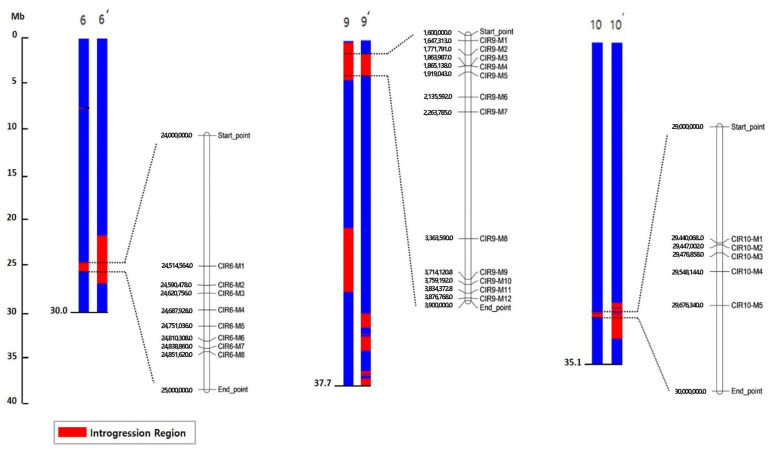
Schematic representation of the three chromosomes (blue) for DRD45NC (left) and DRDSBA (right) showing common introgressed regions (CIRs) (overlapping region in red between DRD45NC and DRDSBA) derived from DRD. The introgressed regions were revealed by an analysis of the single-nucleotide polymorphisms (SNPs) based on whole-genome resequencing. Cleaved amplified polymorphic sequence (CAPS) markers designed from the SNPs in the CIRs and their physical locations (bp) are presented on the right and left sides of each physical map.

The CIRs in the DRD genome, which were found in both DRDSBA and DRD45NC NILs, were detected at approximately 24.0–24.9 Mb on Chr. 6 (CIR-6), 1.6–3.9 Mb on Chr. 9 (CIR-9), and 29.4–30.0 Mb on Chr. 10 (CIR-10) (Figure 3, Figure S2, Table S2). In CIR-6 of DRDSBA and DRD45NC, 897 and 948 SNPs were detected, respectively, including 894 common SNPs in both NILs. In CIR-9 of DRDSBA and DRD45NC, there were 933 and 926 SNPs, respectively, including 919 common SNPs in both NILs. In CIR-10 of DRDSBA and DRD45NC, there were 135 and 363 SNPs, respectively, and only 134 common SNPs in both NILs. Gene annotation information for all SNPs in each CIR is presented in Table S2. The distribution of common SNPs in the two NILs in the genic region is summarized in Table 3, and only these SNPs were considered for the development of cleaved amplified polymorphic sequence (CAPS) markers.

### 2.5. Development of CAPS Markers Associated with HL Contents

**Figure 4:**
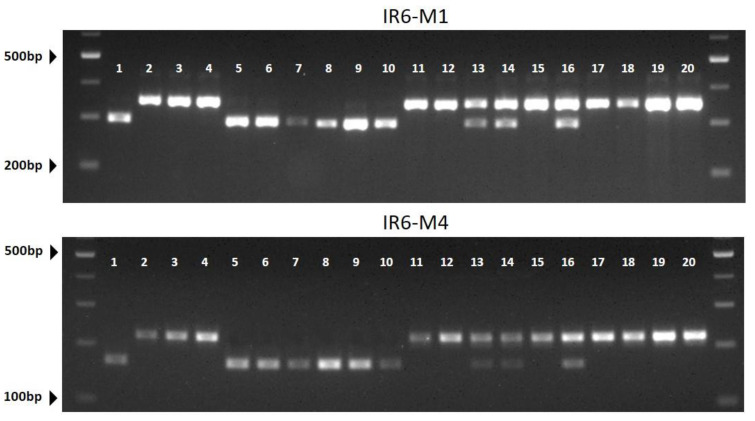
Agarose gel image showing the genotyping results for two cleaved amplified polymorphic sequence (CAPS) markers CIR-M1 and CRI-M4 tested on 20 watermelon cultivars. The number over each lane corresponds to the entry number (EN) for each cultivar shown in Table 1. M, 100-bp size marker.

SNPs in genes in the CIRs were selected to ensure an even genomic distribution, and CAPS markers were designed. A total of 17 SNPs within CIR-6, 18 SNPs in CIR-9, and 6 SNPs in CIR-10 were converted to CAPS markers and evaluated using four parents of the NILs. Unambiguously polymorphic PCR bands were observed for 26 CAPS markers (Table S3 and Figure 3), although PCRs failed or polymorphisms were not observed for the remaining markers. All 20 cultivars were genotyped by using the 26 CAPS markers, and genetic associations between marker genotypes and lycopene contents were evaluated using a single marker analysis and one-way ANOVA (Table 4). The correlation between phenotypes and marker genotypes was the highest for CIR6 (coefficient of correlation (*R*^2^) = 0.46–072 at *p* < 0.05), with the highest values for CIR6-M1 and CIR6-M4 (Table 4 and Figure 4). The *R*^2^ values for the CAPS markers in other CIRs were relatively low at 0.149−0.489, indicating that the major gene(s) responsible for high *trans*-lycopene contents in DRD may be located on CIR6.

### 2.6. Gene Annotation Analysis

The CDS of 16 genes (Table S4) in the watermelon lycopene biosynthesis pathway [21,22,23], namely, geranylgeranyl pyrophosphate (*GGPP*), phytoene synthase (*PSY1*), phytoene desaturase (*PDS*), ζ-carotene desaturase (*ZDS*), carotenoid isomerase (*CRTISO*), lycopene β-cyclase (*LCYB*-cyclase), lycopene α-cyclase (*LCYE*), nine-cis-epoxycarotenoid dioxygenases (*NCED*), β-carotene hydroxylase (*CHYb*), zeaxanthin epoxidase (*ZEP*), and violaxanthin de-epoxidase (*VDE*), were compared among DRD, SBA, and 45NC. The CDS sequences of these genes were highly conserved among the cultivars, and only the following SNPs were found: one nonsynonymous SNP in *PSY1* (Cla97C01G024630), one nonsynonymous and two synonymous SNPs in *PDS1* (Cla97C017G142100), one nonsynonymous and one synonymous SNP in *PDS2* (Cla97C08G151960), and one synonymous SNPs in *NCED* (Cla97C07G137260) (data not shown). Among the three nonsynonymous SNPs, only one in *PDS1* discerned DRD from SBA and 45NC. None of these 11 genes were located in the CIRs, except *PDS* (Cla97C09G165530), which was found in CIR9.

The CIR6 region harbored 106 annotated genes but did not include watermelon lycopene biosynthesis genes. Genes located in the vicinity of two CAPS markers with the highest *R*^2^ values in CIR6 included genes related to ion movement, endopeptidase activity, and lipid and fatty acid biosynthesis.

## 3. Discussion

Lycopenes are major functional compounds in red-fleshed watermelons and have a major influence on fruit quality, such as glucosinolate in Chinese cabbage [24,25], capsaicin in red pepper [26], and quercetin in onion [27,28,29]. Interest in breeding watermelon cultivars with HL contents is increasing [30,31,32] owing to their applications in processed foods, including juice [33], snacks [34], jam [35], and fresh-cut processing, in which nutritional components decrease during distribution and sales [36].

The main lycopene components in watermelon are pro-lycopene (*cis*-lycopene) and *trans*-lycopene, which are abundant in orange-colored flesh and red flesh, respectively [8,37]. Mutations in *CRTISO* are a limiting factor for the conversion of pro-lycopene to *trans*-lycopene and cause the accumulation of pro-lycopene in orange-fleshed watermelon fruits [10]. Red flesh in watermelon is largely divided into scarlet red and coral red, and the former is generally characterized by higher trans-lycopene content than that of the latter [38,39]. Between scarlet red and coral red flesh, most carotenoid synthesis-related genes are expressed in a similar pattern during fruit development phases, except for *LCYB* and *CHYB*. In the early development stage, the expression levels of these two genes increase significantly in scarlet red fruits and remain stable in coral red watermelons [4,21]. However, the genetic regulatory mechanism underlying variation in the lycopene contents is not yet fully understood.

In this study, we identified a genomic region associated with high *trans*-lycopene contents using WGRS of NILs derived from the scarlet red-fleshed breeding line DRD. In a NIL, genes associated with the target trait are likely to exist within the introgressed chromosomal region. However, in the process of backcrossing and selection, certain chromosomal segments not associated with the trait can be transferred incidentally, complicating the genetic analysis. To address this issue, we compared two NILs originating from different recurrent parents to find CIRs. We developed CAPS markers from the SNPs detected in the CIR and evaluated them using 20 cultivars to narrow down a genomic location associated with high *trans*-lycopene contents. Two CAPS markers (CIR6-M1 and CIR6-M4) developed from an introgressed region at 24–25 Mb on Chr. 6 were correlated with the trait.

In a previous study [40], a major QTL (*Y^scr^*) responsible for scarlet red and coral red flesh was mapped to approximately 16.75–23.79 Mb on Chr. 6, which is approximately 300 kb upstream of CIR6. Two InDel markers (InDel27_fc6 and InDel28_fc6) were developed for the candidate genes within the major QTL region [40]. In our study, DRD, SBA, 45NC were genotyped based on these InDel markers. However, the results were inconsistent with flesh colors; DRD showed the marker genotype for coral red, whereas SBA and 45NC showed marker genotypes for scarlet red based on these CAPS (data not shown). We verified the WGRS data for these candidate genes and did not detect sequence variants specific to DRD, indicating that this candidate gene is not involved in the accumulation of *trans*-lycopene in DRD.

Although NILs have the target trait of the donor, they show a nearly identical genomic composition to that of the recurrent parent. Therefore, NILs are suitable for discovering genes or QTLs involved in the target trait by analyses of regions of introgression of the donor’s chromosomal segment [41,42]. Compared to the whole-genome segregating population in plants, such as F2 or recombinant inbred lines, NILs enable the effective identification of the QTLs by minimizing epistatic interactions, which make it difficult to fully define and characterize the effects of individual loci [43]. In watermelon, the NIL population has been used in genetic studies of seed size [44] and fruit cracking [45].

Of the 20 cultivars in this study, the scarlet red-fleshed cultivars showed higher *trans*-lycopene contents than those of the coral red-fleshed cultivars [31,38]. Interestingly, the scarlet red color in the two NILs was similar to that of the donor; however, their *trans*-lycopene contents were intermediate between those of the donor and recurrent parents. Lower *trans*-lycopene contents in NILs than in the donor parent may be because of the fact that genetic factors involved in lycopene accumulation were not fully recovered in these NILs. In addition, we observed that all F1 plants from crosses between scarlet red- and coral red-fleshed cultivars showed substantially lower lycopene contents than that of the HL parental line, indicating that the HL contents of DRD are not controlled by a single dominant or recessive allele. Therefore, differences in the accumulation of *trans*-lycopene between scarlet red and coral red flesh may be determined by a complex genetic regulatory mechanism. Dou et al. [46] revealed that the levels of gibberellin and lycopene accumulation are negatively correlated, whereas there is a positive correlation between the accumulation of ABA and lycopene in the red-fleshed cultivar Mimei.

In conclusion, our WGRS analysis of two NILs and the identification of CIRs revealed the genomic location responsible for the target trait in this study (i.e., *trans*-lycopene contents). Our results indicated that a 3 Mb introgressed region on Chr. 6 is associated with high *trans*-lycopene contents in scarlet red-fleshed watermelon. The CAPS markers developed from this region of introgression were correlated with the accumulation of *trans*-lycopene and can be useful for marker-assisted selection.

## 4. Materials and Methods

### 4.1. Plant Materials

**Figure 5:**
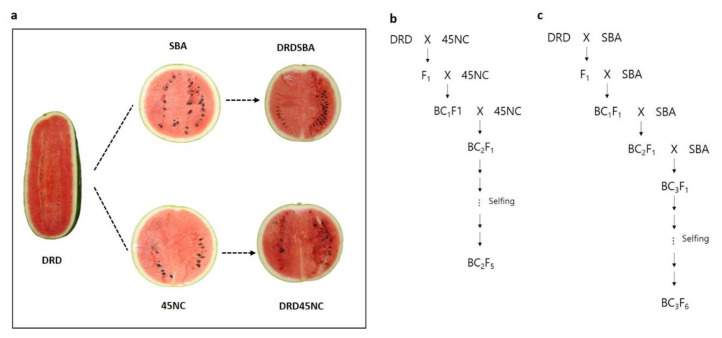
Flesh color of the watermelon cultivar DRD (donor parent, scarlet red), 45NC (recurrent parent, coral red), SBA (recurrent parent, coral red), DRD45NC (near-isogenic line, scarlet red), and DRDSBA (near-isogenic line, scarlet red) (**a**), and the pedigrees of DRD45NC (BC2F5) (**b**) and DRDSBA (BC3F6) (**c**).

For the development of NILs, the scarlet red-fleshed donor parent DRD and three coral red-fleshed recurrent parents, SBA, SBB, and 45NC, were crossed to produce F1 progeny. These three types of F1 hybrids were backcrossed to the recurrent parents to the BC2 or BC3 generation. At each BC generation, the progeny plants with scarlet red flesh were selected for backcrossing. After backcrossing, the progeny were self-pollinated for 2–3 generations to develop three NILs, namely, DRDSBA, DRDSBB, and DRD45NC (Figure 5).

For the analysis of lycopene contents and marker-phenotype association, 20 varieties were used, including the donor parent DRD, 3 recurrent parents SBA, SBB, and 45NC, 3 NILs, 3 scarlet red-fleshed breeding lines, 2 coral red-fleshed breeding lines, 5 experimental F1 hybrids derived from crosses between scarlet red-fleshed inbred lines and coral red-fleshed inbred lines, and 3 coral red-fleshed commercial F1 hybrids (Table 1). DRD, SBA, 45NC, and two NILs (DRDSBA and DRD45NC) were used for NGS-based WGRS.

### 4.2. Planting and Harvesting

Watermelon plants were cultivated from May to August 2018 at a watermelon farm (Changwon, South Korea) using the conventional greenhouse cultivation method to set a single fruit per individual plant. Seeds were sown on a plastic 50-cell tray filled with the bed soil. Seedlings were grafted at the 3–4 true leaf stage with the rootstock Sintoza. At 15 days after grafting, 5–7 healthy seedlings per cultivar were transplanted at 30 cm intervals in the field soil of a plastic film greenhouse. Before transplanting, 2 tons of organic compost and 60 kg of N/P/K were applied to the field soil as a basic fertilizer. During the cultivation period, additional fertilization was performed by applying nitrogen fertilizer and liquid compound fertilizer three times, potassium twice, and phosphoric acid once. Water management was carried out by supplying three tons of water per 500 m^2^ eight times every 7 days. The temperature of the greenhouse was maintained at 35–40 °C during the daytime and 25 °C or higher at night. Pollination was performed by hand for 5 days from 30 days after planting. A single fruit per plant was cultivated and harvested 30–35 days after fertilization. Only fruits that were sufficiently mature were used for the analysis.

### 4.3. Lycopene Contents Analysis

Five fruits were harvested for each cultivar and used for the lycopene contents analysis using high-performance liquid chromatography (HPLC). The central part of the cross-section of the harvested fruit was cut at a thickness of approximately 3 cm, the flesh part was cut into 3 × 3 cm cubes, and the seeds were removed. Then, the flesh samples were put in a zip-lock bag, immediately frozen at −80 °C, and dried with a freeze dryer (VDF0050; Biocryos, Pyeongtaek, Korea).

For sample pretreatment, the freeze-dried pulp was crushed finely, and 0.1 g was added to a 50 mL screw-top tube with beads at the volume ratio of 1:1. Then, 1 mL of ethanol containing 0.5 mM butylated hydroxytoluene was added and shaken vigorously for 2 min and 30 s. The ethanol, samples, and beads in the tube were all transferred to a 15 mL tube, and the tube was washed three times with 1 mL of acetone and transferred to a 15 mL tube. Then, 3 mL of 15 mL petroleum ether was added and vortexed, followed by the addition of 8 mL of 20% NaCl and vortexing. After centrifugation at 3000 rpm for 10 min, only the supernatant was picked up, and after mass-up, Na_2_SO_4_ was added and passed through a filter (PTFE, 13 mm, 0.2 μm; Advantec, USA) to prepare the final analysis sample.

For the quantitative analysis of lycopene, an HPLC system (UltiMate 3000RS; UltiMate, Torrance, CA, USA) equipped with a reverse-phase column (Kinetex 2.6 μm, C18 100A, 100 × 4.60 mm; Phenomenex, Torrance, CA, USA) was used. For mobile phase A, 78% methanol was used, and 100% ethyl acetate was used for phase B. Separation conditions were as follows: 0–8 min, 70% B; 8–10 min, 60% B; 10–12 min, 100% B; 12–14.01 min, 0% B; 14.01–20 min, 100% B, and the flow rate was 1 mL per min. *Trans*-lycopene was used as a standard product for quantification (Sigma-Aldrich, St. Louis, MO, USA), and the contents of lycopene were quantified by measuring absorbance at 289 nm, 450 nm, and 660 nm. The statistical analysis of the lycopene contents was performed using SPSS version 25.0 (IBM SPSS Inc., New York, NY, USA) to verify the differences among the varieties at a 5% significance level using one-way ANOVA and Duncan’s multiple range test.

### 4.4. WGRS Based on NGS

Genomic DNA was extracted from the donor parent DRD, recurrent parents SBA and 45NC, and their two NILs using CTAB from young leaves collected during the seedling period, following the methods described by Hwang et al. [47].The extracted DNA was evaluated using a fluorometer (Qubit; Thermo Fisher Scientific Inc., Waltham, MA, USA) and diluted to a concentration of 30 ng/µL for NGS. Raw reads were generated using paired-end sequencing (2 × 100–150 bp) with the Illumina HiSeq 2000 and NextSeq platforms. As a pre-processing step, only reads with a minimum length of >25 bp and Phred score >20 were filtered using DynamicTrim and LengthSort of SolexaQA (version.1.13) [48]. The selected reads were mapped to the watermelon 97103 genome assembly version 2 (CuGenDB; http://cucurbitgenomics.org) using BWA (0.6.1-r104) [49]. Raw SNPs/InDels were searched using SAMtools (0.1.16) [50], and only biallelic SNPs/InDels with a read depth >3 and mapping quality >30 were filtered out to create an integrated SNP matrix using the SEEDERS in-house script [51]. The filtered SNPs/InDels were genotyped as homozygous or heterozygous based on the proportion (%) of reads supporting one or the other allele (homozygous for proportion ≥90% and heterozygous for 40% ≤ proportion ≤ 60%).

### 4.5. Detection of Introgressed Regions

The introgressed regions in each NIL were detected based on SNPs and InDel loci derived from the donor parent DRD. Only homozygous SNPs satisfying depth ≥ 3 were used for the analysis. The full-length genome sequence of each NIL was divided by a window size of 1 Mb, and the SNP/InDel loci derived from the donor were plotted using GGT2 Graphical Genotypes (Milne et al., 2010). The introgressed regions commonly detected in both NILs were identified as candidate regions harboring gene(s) related to HL contents for further analyses.

### 4.6. Development of Molecular Markers Associated with HL Contents

Homozygous SNPs detected in CIRs and distributed at relatively regular intervals were selected. These SNPs were converted into CAPS markers using their flanking sequences. Primer3 (version. 0.4.0) and NEB Cutter (version 2.0) were used for CAPS marker design.

For CAPS marker genotyping, gDNAs of 20 cultivars were extracted as described in Section 4.4. WGRS based on NGS. PCR was performed in a total volume of 20 µL containing 10 ng of genomic DNA, 0.3 µM each forward and reverse primer, 1× PCR buffer, 0.2 mM dNTPs, and 0.5 U of Taq polymerase (Solgent, Daejeon, Korea). Touch-down PCR was performed under the following conditions: 1 cycle at 95 °C for 5 min, 10 cycles at 95 °C for 15 s, 65 °C (decreased by 0.5 °C for each cycle) for 30 s with the temperature, and 72 °C for 1 min., and 35 cycles of amplification at 95 °C for 15 s, 60 °C for 30 s, and 72 °C for 1 min. The restriction enzyme digestion of the PCR amplicons was performed according to the manufacturer’s protocol (New England BioLabs, Inc., Ipswich, MA, USA). Electrophoresis was performed at 160 V for approximately 1 h and 40 min using a 3% agarose gel in 1× TAE buffer. Gel staining was performed in ethidium bromide, and PCR bands were detected under ultraviolet light.

The *R*^2^ between the marker genotype and lycopene contents was determined using a simple linear regression analysis at the 5% significance level with SPSS (version 25.0).

### 4.7. Gene Annotation

The coding sequences of 16 genes involved in the lycopene biosynthesis pathway were compared between the donor parent DRD and recurrent parents SBA and 45NC. Multiple sequence alignment and allele variant detection were performed using ClustalW. The list of genes located, the region of introgression was annotated based on information for the reference assembly 97103 v2.

## Figures and Tables

**Table 1:** *Trans*-lycopene contents of 20 watermelon cultivars used in this study.

EN.	Cultivar	Generation	Flesh Color	Lycopene Contents (µg/g) ^a^
1	DRD	Inbred	Scarlet red	589.45 ± 71.88e
2	SBA	Inbred	Coral red	225.84 ± 45.06b
3	SBB	Inbred	Coral red	164.04 ± 28.53ab
4	45NC	Inbred	Coral red	213.47 ± 28.25b
5	DRDSBA	NIL (BC3F6)	Scarlet red	477.69 ± 46.92d
6	DRDSBB	NIL (BC3F9)	Scarlet red	369.60 ± 44.79c
7	DRD45NC	NIL (BC2F5)	Scarlet red	439.04 ± 38.78d
8	NS514	Inbred	Scarlet red	463.13 ± 72.55d
9	NS516	Inbred	Scarlet red	689.17 ± 100.29f
10	DRF5819	Inbred	Scarlet red	496.16 ± 44.69d
11	52192	Inbred	Coral red	213.16 ± 64.66b
12	Pink Variety	Inbred	Coral red	191.05 ± 33.83ab
13	DRDSBA × SBA	Experimental F1	Coral red	193.43 ± 25.46ab
14	DRDSBA × NS514	Experimental F1	Coral red	213.73 ± 39.57b
15	DRHS4105 × 52192	Experimental F1	Coral red	203.17 ± 20.92ab
16	DRF5819 × 52192	Experimental F1	Coral red	142.64 ± 14.76a
17	SBA × 52192	Experimental F1	Coral red	189.62 ± 46.04ab
18	Dangdanghan	Commercial F1	Coral red	135.77 ± 18.07a
19	Chamjoeun	Commercial F1	Coral red	189.79 ± 29.12ab
20	Bigstart	Commercial F1	Coral red	211.99 ± 35.02b

a—The letters indicate statistically significant differences of trans-lycopene contents of watermelon between cultivars based on the one-way ANOVA and Duncan’s multiple range tests.

**Table 2:** Summary of the trimmed sequencing data for parental lines and near-isogenic lines.

Samples	No. of Reads	Total Length (bp)	Avg. Length	Trimmed/Raw(%)	Genome CoverAge (×) ^a^
DRD	58,036,656	5,382,805,154	92.75	79.24	24.76
	58,036,656	5,140,713,545	88.58	75.68	
SBA	67,875,107	6,288,288,055	92.64	79.72	29.03
	67,875,107	6,049,639,852	89.13	76.70	
45NC	58,499,614	5,428,904,346	92.80	81.46	25.02
	58,499,614	5,206,666,818	89.00	78.12	
DRDSBA	78,305,938	7,244,678,852	92.52	78.89	33.40
	78,305,938	6,951,305,936	88.77	75.69	
DRD45NC	57,130,281	5,298,242,278	92.74	81.88	24.44
	57,130,281	5,090,776,571	89.11	78.68	

^a^—The numbers indicate approximate genome coverage.

**Table 3:** Genomic information of the common introgressed region (CIR) in the two near-isogenic lines and the single-nucleotide polymorphisms (SNPs) revealed from the CIRs.

CIR	Location		No. of Genes	SNP			
	Chr.	Mb		Promoter ^a^	Exon	Intron	Total
CIR6	6	24.06–24.99	1031	105	42	111	909
CIR9	9	1.60–3.96	3171	160	61	200	960
CIR10	10	29.38–29.97	401	14	5	44	138

^a^—The 1kb upstream sequence from the start position of each gene was defined as the promoter region.

**Table 4:** Cleaved amplified polymorphic sequence (CAPS) marker genotyping results for 20 cultivars and correlation coefficients (*R^2^*) for the relationships between the marker genotype and trans-lycopene contents.

Marker ID	Cultivars ^a^																				*R* ^2^
	1	2	3	4	5	6	7	8	9	10	11	12	13	14	15	16	17	18	19	20	
CIR6-M1	hh	ll	ll	ll	hh	hh	hh	hh	hh	hh	ll	ll	hl	hl	ll	hl	ll	ll	ll	ll	0.722
CIR6-M2	hh	ll	ll	ll	hh	hh	hh	hh	hh	hh	hh	ll	hl	hl	hl	hh	hl	ll	ll	ll	0.461
CIR6-M3	hh	ll	ll	ll	hh	hh	hh	hh	hh	hh	hh	ll	hl	hl	hl	hh	hl	ll	ll	ll	0.461
CIR6-M4	hh	ll	ll	ll	hh	hh	hh	hh	hh	hh	ll	ll	hl	hl	ll	hl	ll	ll	ll	ll	0.722
CIR6-M5	hh	ll	ll	ll	hh	hh	hh	hh	hh	hh	hh	ll	hl	hl	hl	hh	hl	ll	ll	ll	0.461
CIR6-M6	hh	ll	ll	ll	hh	hh	hh	hh	hh	hh	hh	ll	hl	hl	hl	hh	hl	ll	ll	ll	0.461
CIR6-M7	hh	ll	ll	ll	hh	hh	hh	hh	hh	hh	hh	ll	hl	hl	hl	hh	hl	ll	hl	ll	0.446
CIR6-M8	hh	ll	ll	ll	hh	hh	hh	hh	hh	hh	hh	ll	hl	ll	hl	hh	ll	ll	hl	ll	0.472
CIR9-M1	hh	ll	ll	ll	hh	ll	hh	hh	hh	hh	hh	ll	hl	hh	hl	hh	hl	ll	hl	ll	0.316
CIR9-M2	hh	ll	ll	ll	hh	ll	hh	hh	hh	hh	ll	ll	hl	hh	hl	hl	hh	hl	m	hh	0.295
CIR9-M3	hh	ll	ll	ll	hh	ll	hh	hh	hh	ll	ll	ll	hl	hh	ll	ll	ll	hh	hl	ll	0.284
CIR9-M4	hh	ll	ll	ll	hh	ll	hh	hh	hh	ll	ll	ll	hl	hh	ll	ll	ll	ll	ll	ll	0.480
CIR9-M5	hh	ll	ll	ll	hh	ll	hh	hh	hh	hh	ll	ll	hl	hh	hl	hl	ll	hl	hl	hl	0.449
CIR9-M6	hh	ll	ll	ll	hh	ll	hh	hh	hh	ll	ll	ll	hl	hh	hl	ll	ll	hl	hl	hh	0.268
CIR9-M7	hh	ll	ll	ll	hl	ll	hh	hh	hh	ll	ll	ll	hl	hh	hl	ll	ll	hl	hl	hh	0.226
CIR9-M8	hh	ll	ll	ll	hh	ll	hh	hh	hh	hh	hh	ll	hl	hh	hh	hh	hl	hl	hh	hh	0.149
CIR9-M9	hh	ll	ll	ll	hh	ll	hh	hh	hl	hh	hl	ll	ll	hh	hl	hh	hl	ll	hl	ll	0.258
CIR9-M10	hh	ll	ll	ll	hh	ll	hh	hh	hh	ll	hh	ll	hl	hh	hl	hl	hl	ll	ll	ll	0.269
CIR9-M11	hh	ll	ll	ll	hh	ll	hh	hh	hh	hh	hh	ll	hl	hh	hl	hh	hl	ll	hl	ll	0.316
CIR9-M12	hh	ll	ll	ll	hh	ll	hh	hh	hh	hh	m	ll	hl	hh	hl	hh	hl	ll	hl	ll	0.356
CIR9-M13	hh	ll	ll	ll	hh	ll	hh	hh	hh	hh	hh	ll	hl	hh	hl	hh	hl	ll	hl	ll	0.316
CIR10-M1	hh	ll	ll	ll	hh	ll	hh	hh	hh	ll	ll	ll	hl	hh	ll	ll	ll	ll	hh	ll	0.356
CIR10-M2	hh	ll	ll	ll	hh	ll	hh	hh	hh	ll	hh	ll	hl	hh	hl	hl	hl	ll	hh	ll	0.193
CIR10-M3	hh	ll	ll	ll	d	ll	ll	d	d	ll	ll	ll	d	d	ll	ll	ll	ll	d	ll	-
CIR10-M4	hh	ll	ll	ll	d	ll	d	d	d	ll	ll	ll	d	d	ll	ll	ll	ll	d	ll	-
CIR10-M5	hh	ll	ll	ll	hh	ll	hh	hh	hh	ll	ll	ll	hl	hh	ll	ll	hl	m	hh	m	0.329

^a^—Arabic numbers correspond to the entry number (EN) for each cultivar shown in Table 1. Hh—homozygous marker genotype for DRD (high *trans*-lycopene); ll—homozygous marker genotype for SBA and 45NC (low *trans*-lycopene); hl—heterozygous marker genotype; d—dominant marker genotype for DRD (marker genotype for DRD and heterozygosity could not be discerned); m—failed PCR.

## Data Availability

The NGS reads data for DRD (SRA submission no.: SRR17070236), SBA (SRR17070232), 45NC (SRR17070234), DRDSBA (SRR17070233), and DRD45NC (SRR17070235) presented in this study are openly available in the National Center for Biotechnology Information (Bioproject: PRJNA783438). The vcf files for SNP information is also provided upon request.

## Supplementary Materials

The following are available online at https://www.mdpi.com/article/10.3390/plants11010008/s1, Table S1: Lycopene contents of 20 watermelon cultivars, Table S2: Single-nucleotide polymorphisms (SNPs) in three 3 common introgressed regions (CIRs), Table S3: Cleaved amplified polymorphic sequence (CAPS) markers developed from the common introgression regions in two near-isogenic lines. Table S4: List of 16 genes involved in the lycopene biosynthesis pathway Figure S1: The number and genomic distribution of the single-nucleotide polymorphisms (SNPs) on each chromosome, Figure S2: Introgressed regions in DRD45NC and DRDSBA detected based on the frequencies of single-nucleotide polymorphisms (SNPs).

## References

[1] Akashi K., Mifune Y., Morita K., Ishitsuka S., Tsujimoto H., Ishihara T. (2017). Spatial accumulation pattern of citrulline and other nutrients in immature and mature watermelon fruits. J. Sci. Food Agric..

[2] Bhandari S., Cho M.-C., Lee J.G. (2016). Genotypic variation in carotenoid, ascorbic acid, total phenolic, and flavonoid contents, and antioxidant activity in selected tomato breeding lines. Hortic. Environ. Biotechnol..

[3] Mohanty N.K., Saxena S., Singh U.P., Goyal N.K., Arora R.P. (2005). Lycopene as a chemopreventive agent in the treatment of high-grade prostate intraepithelial neoplasia. Urol. Oncol..

[4] Zhao W., Lv P., Gu H. (2013). Studies on carotenoids in watermelon flesh. Agric. Sci..

[5] Liu C., Zhang H., Dai Z., Liu X., Liu Y., Deng X., Chen F., Xu J. (2012). Volatile chemical and carotenoid profiles in watermelons [*Citrullus vulgaris* (Thunb.) Schrad (Cucurbitaceae)] with different flesh colors. Food Sci. Biotechnol..

[6] Bang H., Kim S., Leskovar D., King S. (2005). Genotype analysis of fruit color using a molecular marker in watermelon [*Citrullus lanatus* (Thunb.) Matsum & Nakai]. HortScience.

[7] Tadmor Y., King S., Levi A., Davis A., Meir A., Wasserman B., Hirschberg J., Lewinsohn E. (2005). Comparative fruit colouration in watermelon and tomato. Food Res. Int..

[8] Grassi S., Piro G., Lee J., Zheng Y., Fei Z., Dalessandro G., Giovannoni J., Lenucci M. (2013). Comparative genomics reveals candidate carotenoid pathway regulators of ripening watermelon fruit. BMC Genom..

[9] Gusmini G., Wehner T. (2006). Qualitative inheritance of rind pattern and flesh color in watermelon. J. Hered..

[10] Jin B., Lee J., Kweon S., Cho Y., Choi Y., Lee S.J., Park Y. (2019). Analysis of flesh color-related carotenoids and development of a CRTISO gene-based DNA marker for prolycopene accumulation in watermelon. Hortic. Environ. Biotechnol..

[11] Branham S., Vexler L., Meir A., Tzuri G., Frieman Z., Levi A., Wechter W., Tadmor Y., Gur A. (2017). Genetic mapping of a major codominant QTL associated with β-carotene accumulation in watermelon. Mol. Breed..

[12] Bang H., Kim S., Leskovar D., King S. (2007). Development of a codominant CAPS marker for allelic selection between canary yellow and red watermelon based on SNP in lycopene β-cyclase (LCYB) gene. Mol. Breed..

[13] Glover K., Wang D., Arelli P., Carlson S., Cianzio S., Diers B.W. (2004). Near isogenic lines confirm a soybean cyst nematode resistance gene from PI 88788 on linkage group J. Crop. Sci..

[14] Maughan J., Maroof S., Buss G., Huestis G. (1996). Amplified fragment length polymorphism (AFLP) in soybean: Species diversity, inheritance, and near-isogenic line analysis. Theor. Appl. Genet..

[15] Samsampour D., Zanjani B., Jk P., Singh A., Charpe A., Gupta S., Prabhu K. (2010). Identification of molecular markers linked to adult plant leaf rust resistance gene *Lr48* in wheat and detection of *Lr48* in the thatcher near-isogenic line with gene *Lr25*. Euphytica.

[16] Muehlbauer G., Specht J., Staswick P., Graef G., Thomas-Compton M. (1989). Application of the near-isogenic line gene mapping technique to isozyme markers. Crop. Sci..

[17] Gao L., Zhao S., Lu X., He N., Zhu H., Dou J., Wenge L. (2018). Comparative transcriptome analysis reveals key genes potentially related to soluble sugar and organic acid accumulation in watermelon. PLoS ONE.

[18] Han B.K., Rhee S.J., Jang Y.J., Sim T.Y., Kim Y.J., Park T.S., Lee G. (2016). Identification of a causal pathogen of watermelon powdery mildew in Korea and development of a genetic linkage marker for resistance in watermelon (*Citrullus lanatus*). Hortic. Sci. Technol..

[19] Varshney R., Hoisington D., Nayak S., Graner A. (2009). Molecular plant breeding: Methodology and achievements. Plant. Genome.

[20] Voelkerding K., Dames S., Durtschi J. (2010). Next generation sequencing for clinical diagnostics-principles and application to targeted resequencing for hypertrophic cardiomyopathy: A paper from the 2009 william beaumont hospital symposium on molecular pathology. J. Mol. Diagn..

[21] Kang B., Zhao W.E., Hou Y., Tian P. (2010). Expression of carotenogenic genes during development and ripening of watermelon fruit. Sci. Hortic..

[22] Liu G., Yang X., Xu J., Zhang M., Hou Q., Zhu L., Huang Y., Xiong A.-S. (2016). Morphological observation, RNA-Seq quantification, and expression profiling: Novel insight into grafting-responsive carotenoid biosynthesis in watermelon grafted onto pumpkin rootstock. Acta Biochim. Biophys. Sin..

[23] Lv P., Li N., Liu H., Gu H., Zhao W.E. (2014). Changes in carotenoid profiles and in the expression pattern of the genes in carotenoid metabolisms during fruit development and ripening in four watermelon cultivars. Food Chem..

[24] Traka M., Mithen R. (2009). Glucosinolates, isothiocyanates and human health. Phytochem. Rev..

[25] Chen X., Zhu Z., Gerendas J., Zimmermann N. (2008). Glucosinolates in chinese *Brassica campestris* vegetables: Chinese cabbage, purple cai-tai, choysum, pakchoi, and turnip. HortScience.

[26] Saleh B.K., Omer A., Teweldemedhin B. (2018). Medicinal uses and health benefits of chili pepper (*Capsicum* spp.): A review. MOJ Food Process. Technol..

[27] Kumar R., Subramanian V., Nadanasabapathi S. (2017). Health benefits of quercetin. Def. Life Sci. J..

[28] Griffiths G., Trueman L., Crowther T., Thomas B., Smith B. (2002). Onions? A global benefit to health. Phytother. Res..

[29] Jan A.T., Kamli M., Murtaza I., Singh J., Ali A., Haq Q. (2010). Dietary flavonoid quercetin and associated health benefits—An overview. Food Rev. Int..

[30] Perkins P., Davis A. (2004). In search of high lycopene watermelon. Rep. Cucurbit Genet. Coop..

[31] Wang C., Qiao A., Fang X., Sun L., Gao P., Davis A., Liu S., Luan F. (2019). Fine mapping of lycopene content and flesh color related gene and development of molecular marker–assisted selection for flesh color in watermelon (*Citrullus lanatus*). Front. Plant. Sci..

[32] Edwards A., Vinyard B., Wiley E., Brown E., Collins J., Perkins P., Baker R., Clevidence B. (2003). Consumption of watermelon juice increases plasma concentrations of lycopene and β-carotene in humans. J. Nutr..

[33] Via F., Gomes S., Costa P., Campos M., Tonon R., Couri S., Cabral L. (2013). Watermelon juice pretreatment with microfiltration process for obtaining lycopene. Food Sci. Technol. Int..

[34] Maoto M., Beswa D., Jideani A. (2019). Watermelon as a potential fruit snack. Int. J. Food Prop..

[35] Sreelakshmi A., Vidhya D. (2020). Preparation of nutrient rich jam using watermelon (*Citrullus Lanatus*) and hibiscus extract. IJPRSE.

[36] Perkins P., Collins J. (2004). Flesh quality and lycopene stability of fresh-cut watermelon. Postharvest Biol. Technol..

[37] Lewinsohn E., Sitrit Y., Azulay Y., Meir A., Zamir D., Tadmor Y. (2005). Carotenoid pigmentation affects the volatile composition of tomato and watermelon fruits, as revealed by comparative genetic analyses. J. Agric. Food Chem..

[38] Wehner T., Naegele R., Perkins P. (2017). Heritability and genetic variance components associated with citrulline, arginine, and lycopene content in diverse watermelon cultigens. HortScience.

[39] Perkins P., Collins J., Pair S., Roberts W. (2001). Lycopene content differs among red-fleshed watermelon cultivars. J. Sci. Food Agric..

[40] Li N., Shang J., Wang J., Zhou D., Li N., Ma S. (2020). Discovery of the genomic region and candidate genes of the scarlet red flesh color (*Yscr*) locus in watermelon (*Citrullus lanatus* L.). Front. Plant. Sci..

[41] Collard B., Cruz C., McNally K., Virk P., Mackill D. (2008). Rice molecular breeding laboratories in the genomics era: Current status and future considerations. Int. J. Plant. Genom..

[42] Pumphrey M., Bernardo R., Anderson J.A. (2007). Validating the QTL for fusarium head blight resistance in near-isogenic wheat lines developed from breeding populations. Crop. Sci..

[43] Fletcher R., Mullen J., Yoder S., Bauerle W., Reuning G., Sen S., Meyer E., Juenger T., McKay J. (2013). Development of a next-generation NIL library in arabidopsis thaliana for dissecting complex traits. BMC Genom..

[44] Kim Y.-J., Yang T.-J., Park Y.-H., Lee Y.-J., Kang S.-C., Kim Y.-K., Cho J.-L. (2009). Development of near isogenic lines with various seed sizes and study on seed size-related characteristics in watermelon. Korean J. Breed. Sci..

[45] Jiang H., Tian H., Yan C., Jia L., Wang Y., Wang M., Jiang C.J., Li Y., Jiang J., Fang L. (2019). RNA-seq analysis of watermelon (*Citrullus lanatus*) to identify genes involved in fruit cracking. Sci Hortic.

[46] Dou J.-L., Yuan P.-L., Zhao S.-J., He N., Zhu H.-J., Gao L., Ji W.-l., Lu X.-Q., Liu W.-G. (2017). Effect of ploidy level on expression of lycopene biosynthesis genes and accumulation of phytohormones during watermelon (*Citrullus lanatus*) fruit development and ripening. J. Integr Agric..

[47] Hwang J.-H., Park Y.-O., Kim S.-C., Lee Y.-J., Kang J.-S., Choi Y.-W., Son B.-G., Park Y.-H. (2010). Evaluation of genetic diversity among persimmon cultivars (Diospyros kaki Thunb.) using microsatellite markers. J. Life Sci..

[48] Cox M.P., Peterson D.A., Biggs P.J. (2010). SolexaQA: At-a-glance quality assessment of Illumina second-generation sequencing data. BMC Bioinform..

[49] Li H., Durbin R. (2009). Fast and accurate short read alignment with Burrows–Wheeler transform. Bioinformatics.

[50] Li H., Handsaker B., Wysoker A., Fennell T., Ruan J., Homer N., Marth G., Abecasis G., Durbin R., Subgroup G.P.D.P. (2009). The Sequence Alignment/Map format and SAMtools. Bioinformatics.

[51] Kim J.-E., Oh S.-K., Lee J.-H., Lee B.-M., Jo S.-H. (2014). Genome-wide SNP calling using next generation sequencing data in tomato. Mol. Cells.

