# Supplementary material for: Whole-Genome Resequencing of Near-Isogenic Lines Reveals a Genomic Region Associated with High Trans-Lycopene Contents in Watermelon
Source: Plants (Basel). 2021 Dec 21;11(1):8. doi: 10.3390/plants11010008 (PMC8747524; doi:10.3390/plants11010008)

Figure S1: The number and genomic distribution of the SNPs between the donor DRD and recurrent parent SBA (A) and 45NC (B) on each chromosome

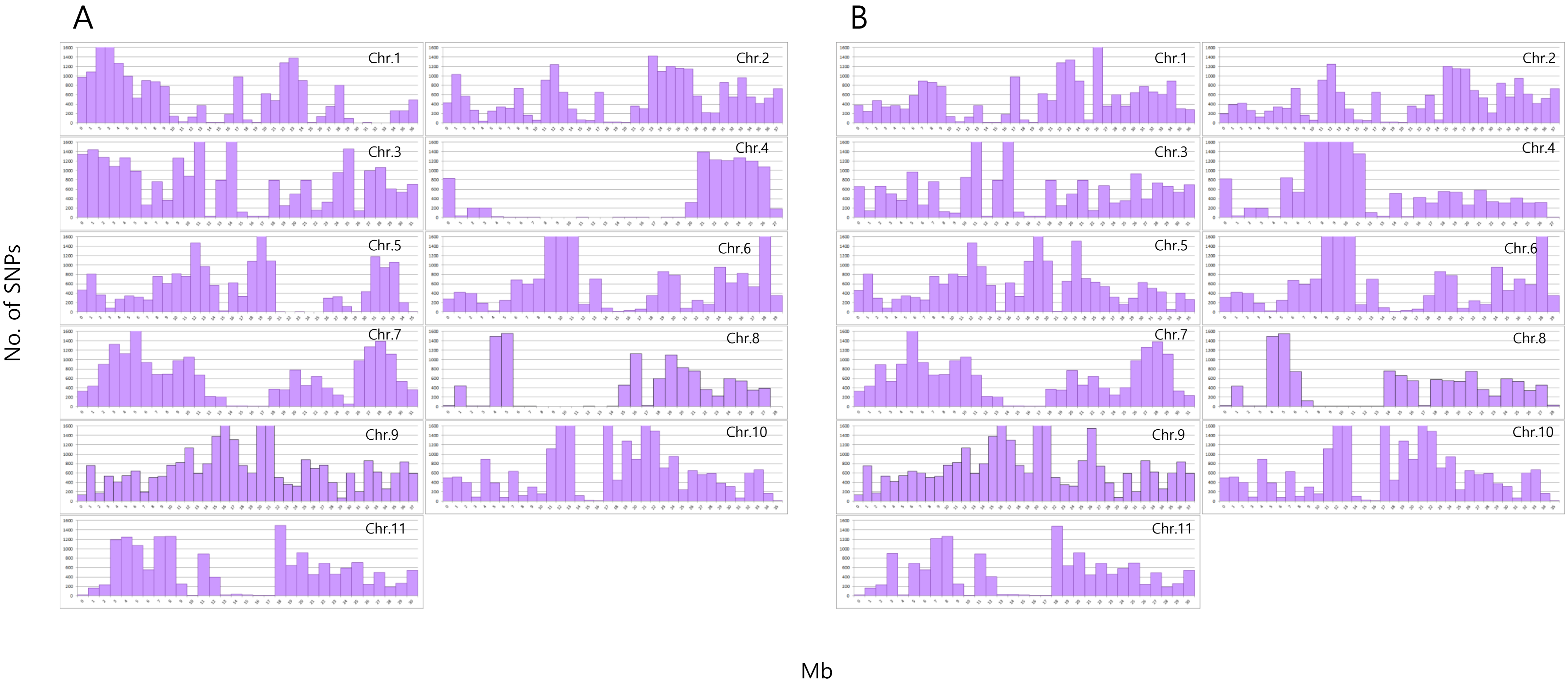

Supplement: Supplementary file 1 [file plants-11-00008-s001.zip › Figure S1.pdf]
