# Supplementary material for: Whole-Genome Resequencing of Near-Isogenic Lines Reveals a Genomic Region Associated with High Trans-Lycopene Contents in Watermelon
Source: Plants (Basel). 2021 Dec 21;11(1):8. doi: 10.3390/plants11010008 (PMC8747524; doi:10.3390/plants11010008)
Supplement: Supplementary file 1 [file plants-11-00008-s001.zip › Table S1.pdf]

Table S1: Lycopene contents of 20 watermelon cultivars

Name	lycopene(μg/g)
1	690.68
1	497.52
1	552.55
1	613.85
1	592.66
Average	589.45
standard deviation	71.88

Name	lycopene(μg/g)
2	250.81
2	173.26
2	248.23
2	274.36
2	182.52
Average	225.84
standard deviation	45.06

Name
3
3
3
3
3
Average
standard deviation

Name	lycopene(μg/g)
7	475.45
7	377.16
7	460.35
7	455.21
7	427.01
Average	439.04
standard deviation	38.78

Name	lycopene(μg/g)
8	549.53
8	406.13
8	395.46
8	432.13
8	532.42
Average	463.13
standard deviation	72.55

Name
9
9
9
9
9
Average
standard deviation

Name	lycopene(μg/g)
13	188.36
13	202.85
13	174.48
13	232.45
13	169.00
Average	193.43
standard deviation	25.46

Name	lycopene(μg/g)
14	256.01
14	220.06
14	245.91
14	165.14
14	181.51
Average	213.73
standard deviation	39.57

Name
15
15
15
15
15
Average
standard deviation

Name	lycopene(μg/g)
19	183.24
19	202.35
19	175.55
19	155.48
19	232.35
Average	189.79
standard deviation	29.12

Name	lycopene(μg/g)
20	225.34
20	205.30
20	183.45
20	265.54
20	180.34
Average	211.99
standard deviation	35.02

lycopene(μg/g)
149.60
179.04
130.55
156.46
204.57
164.04
28.53

Name	lycopene(μg/g)
4	240.74
4	213.30
4	179.60
4	191.59
4	242.15
Average	213.47
standard deviation	28.25

Name	lycopene(μg/g)
5	498.87
5	456.29
5	439.05
5	550.29
5	443.96
Average	477.69
standard deviation	46.92

lycopene(μg/g)
546.62
638.13
699.78
795.64
765.68
689.17
100.29

Name	lycopene(μg/g)
10	430.79
10	498.71
10	556.93
10	494.45
10	499.93
Average	496.16
standard deviation	44.69

Name	lycopene(μg/g)
11	168.16
11	209.36
11	177.38
11	325.55
11	185.36
Average	213.16
standard deviation	64.66

lycopene(μg/g)
182.98
218.21
230.89
185.07
198.69
203.17
20.92

Name	lycopene(μg/g)
16	134.52
16	133.64
16	138.97
16	168.77
16	137.28
Average	142.64
standard deviation	14.76

Name	lycopene(μg/g)
17	124.36
17	230.08
17	210.86
17	193.18
17	158.24
Average	189.62
standard deviation	46.04

Name	lycopene(μg/g)
6	342.24
6	316.47
6	399.77
6	361.04
6	428.50
Average	369.60
standard deviation	44.79

Name	lycopene(μg/g)
12	174.01
12	166.08
12	250.32
12	182.36
12	182.45
Average	191.05
standard deviation	33.83

Name	lycopene(μg/g)
18	117.13
18	150.01
18	133.34
18	120.11
18	158.24
Average	135.77
standard deviation	18.07
