# Supplementary material for: Whole-Genome Resequencing of Near-Isogenic Lines Reveals a Genomic Region Associated with High Trans-Lycopene Contents in Watermelon
Source: Plants (Basel). 2021 Dec 21;11(1):8. doi: 10.3390/plants11010008 (PMC8747524; doi:10.3390/plants11010008)
Supplement: Supplementary file 1 [file plants-11-00008-s001.zip › Table S2.pdf]

Table S2: Single Nucleotide Polymorphisms (SNPs) in three 3 common introgressed regions (CIRs)

Chromosome	pos	ref	DRD	DRD_depth	DRD45NC	DRDSBA	DRD45NC_depth	45NC
Cla97Chr10	29385009	A	A	22|22	A	A	22|22	T
Cla97Chr10	29419696	A	A	17|18	A	A	18|18	T
Cla97Chr10	29425958	T	T	15|15	T	T	29|29	C
Cla97Chr10	29427454	A	A	19|19	A	A	19|19	G
Cla97Chr10	29428130	A	A	26|26	A	A	23|23	G
Cla97Chr10	29428358	G	G	16|16	G	G	29|29	A
Cla97Chr10	29431146	G	G	14|14	G	G	19|19	A
Cla97Chr10	29440068	G	G	17|17	G	G	25|25	T
Cla97Chr10	29442713	A	A	24|24	A	A	19|19	G
Cla97Chr10	29442794	G	G	33|33	G	G	21|21	A
Cla97Chr10	29443115	G	G	23|23	G	G	17|17	C
Cla97Chr10	29443243	A	A	14|14	A	A	17|17	G
Cla97Chr10	29444589	T	T	12|12	T	T	25|25	C
Cla97Chr10	29445010	A	A	26|26	A	A	15|16	T
Cla97Chr10	29447001	T	T	12|12	T	T	31|31	C
Cla97Chr10	29447460	C	C	14|14	C	C	28|29	T
Cla97Chr10	29452466	A	A	17|17	A	A	21|21	T
Cla97Chr10	29453166	C	C	20|20	C	C	27|27	T
Cla97Chr10	29453946	G	G	17|17	G	G	27|27	A
Cla97Chr10	29454665	T	T	19|19	T	T	22|22	A
Cla97Chr10	29455194	C	C	18|18	C	C	33|33	A
Cla97Chr10	29455285	C	C	19|19	C	C	26|26	A
Cla97Chr10	29456074	G	G	18|18	G	G	26|26	A
Cla97Chr10	29456093	A	A	19|19	A	A	23|23	G
Cla97Chr10	29456508	A	A	16|16	A	A	28|28	G
Cla97Chr10	29457583	G	G	18|18	G	G	15|15	T
Cla97Chr10	29457926	C	C	15|15	C	C	27|27	T
Cla97Chr10	29457999	T	T	13|13	T	T	28|28	A
Cla97Chr10	29458292	G	G	17|17	G	G	32|32	A
Cla97Chr10	29459085	T	T	22|22	T	T	27|27	C
Cla97Chr10	29459565	G	G	19|19	G	G	34|34	A
Cla97Chr10	29459744	T	T	16|16	T	T	25|25	C
Cla97Chr10	29460107	G	G	13|13	G	G	17|17	T
Cla97Chr10	29460801	A	A	7|7	A	A	16|16	G
Cla97Chr10	29460810	A	A	7|7	A	A	17|17	G
Cla97Chr10	29460826	A	A	8|8	A	A	14|14	G
Cla97Chr10	29461089	C	C	7|7	C	C	21|21	A
Cla97Chr10	29461181	C	C	17|17	C	C	25|25	T
Cla97Chr10	29461369	T	T	20|20	T	T	26|26	C
Cla97Chr10	29461632	T	T	20|20	T	T	28|31	C
Cla97Chr10	29461878	C	C	21|21	C	C	29|29	A
Cla97Chr10	29461945	C	C	21|21	C	C	25|25	T
Cla97Chr10	29462031	C	C	14|14	C	C	32|32	T
Cla97Chr10	29462275	T	T	13|13	T	T	24|24	A
Cla97Chr10	29462519	C	C	19|19	C	C	29|29	T
Cla97Chr10	29462748	C	C	20|20	C	C	31|31	T
Cla97Chr10	29463298	C	C	18|18	C	C	32|32	T

Cla97Chr10	29463963	A	A	27|27	A	A	32|32	G
Cla97Chr10	29464899	C	C	18|18	C	C	22|22	A
Cla97Chr10	29465058	C	C	28|28	C	C	32|32	T
Cla97Chr10	29466918	G	G	23|23	G	G	22|22	A
Cla97Chr10	29466965	T	T	22|22	T	T	27|27	A
Cla97Chr10	29467685	A	A	21|21	A	A	34|34	G
Cla97Chr10	29468398	T	T	15|15	T	T	23|23	A
Cla97Chr10	29468454	C	C	24|24	C	C	23|23	T
Cla97Chr10	29468469	T	T	25|25	T	T	24|24	C
Cla97Chr10	29470884	C	C	22|22	C	C	18|18	A
Cla97Chr10	29470890	T	T	21|21	T	T	18|18	C
Cla97Chr10	29473197	C	C	19|19	C	C	23|23	T
Cla97Chr10	29476334	T	T	18|18	T	T	23|23	C
Cla97Chr10	29476855	G	G	21|21	G	G	37|37	A
Cla97Chr10	29479816	A	A	18|18	A	A	30|30	G
Cla97Chr10	29481002	A	A	27|27	A	A	33|33	T
Cla97Chr10	29481030	C	C	24|24	C	C	26|26	T
Cla97Chr10	29530475	T	T	18|18	T	T	36|36	C
Cla97Chr10	29533171	G	G	16|16	G	G	11|11	A
Cla97Chr10	29537693	G	G	21|21	G	G	18|18	A
Cla97Chr10	29538043	G	G	33|33	G	G	26|26	C
Cla97Chr10	29548144	A	A	18|18	A	A	27|27	T
Cla97Chr10	29556663	G	G	27|27	G	G	44|44	A
Cla97Chr10	29557773	A	A	13|13	A	A	21|21	T
Cla97Chr10	29569235	A	A	24|24	A	A	24|24	G
Cla97Chr10	29570254	C	C	21|21	C	C	17|17	T
Cla97Chr10	29570514	G	G	31|31	G	G	21|21	A
Cla97Chr10	29580134	A	A	14|15	A	A	21|21	T
Cla97Chr10	29580772	A	A	15|15	A	A	20|20	T
Cla97Chr10	29582180	T	T	14|14	T	T	23|23	C
Cla97Chr10	29586177	C	C	17|17	C	C	25|25	T
Cla97Chr10	29586628	T	T	22|22	T	T	23|23	C
Cla97Chr10	29587047	T	T	22|22	T	T	12|12	C
Cla97Chr10	29592118	T	T	16|16	T	T	28|28	A
Cla97Chr10	29592764	A	A	27|27	A	A	25|25	T
Cla97Chr10	29596687	T	T	10|10	T	T	14|14	C
Cla97Chr10	29602266	C	C	24|24	C	C	26|26	A
Cla97Chr10	29612076	C	C	29|29	C	C	31|31	A
Cla97Chr10	29612999	G	G	19|19	G	G	19|19	T
Cla97Chr10	29616384	A	A	31|31	A	A	29|29	T
Cla97Chr10	29634487	A	A	22|22	A	A	19|19	G
Cla97Chr10	29634777	G	G	22|22	G	G	20|20	A
Cla97Chr10	29642692	A	A	21|21	A	A	27|27	G
Cla97Chr10	29663135	G	G	9|9	G	G	27|27	A
Cla97Chr10	29663323	C	C	18|18	C	C	24|24	A
Cla97Chr10	29667313	T	T	12|12	T	T	27|27	C
Cla97Chr10	29667399	G	G	13|13	G	G	25|25	A
Cla97Chr10	29667829	T	T	14|14	T	T	15|15	C
Cla97Chr10	29667931	A	A	18|18	A	A	15|15	G
Cla97Chr10	29668814	A	A	22|22	A	A	36|36	C

Cla97Chr10	29668826	A	A	22|22	A	A	30|30	G
Cla97Chr10	29668996	A	A	18|18	A	A	31|31	G
Cla97Chr10	29670535	T	T	31|31	T	T	38|38	G
Cla97Chr10	29670723	C	C	22|22	C	C	17|17	T
Cla97Chr10	29670872	T	T	22|22	T	T	33|33	A
Cla97Chr10	29670962	A	A	28|28	A	A	41|41	T
Cla97Chr10	29671708	T	T	20|20	T	T	20|20	A
Cla97Chr10	29675580	G	G	5|5	G	G	8|8	A
Cla97Chr10	29676339	T	T	21|21	T	T	24|24	C
Cla97Chr10	29677836	A	A	9|9	A	A	8|8	T
Cla97Chr10	29678169	C	C	25|25	C	C	15|16	T
Cla97Chr10	29682757	C	C	13|13	C	C	18|18	T
Cla97Chr10	29686212	T	T	19|19	T	T	22|22	C
Cla97Chr10	29689363	G	G	20|20	G	G	32|32	A
Cla97Chr10	29691236	A	A	25|25	A	A	13|13	T
Cla97Chr10	29693902	C	C	32|32	C	C	33|33	T
Cla97Chr10	29696337	T	T	21|21	T	T	37|37	G
Cla97Chr10	29696965	A	A	26|26	A	A	25|25	C
Cla97Chr10	29701102	T	T	28|28	T	T	34|34	C
Cla97Chr10	29701427	G	G	35|35	G	G	25|25	T
Cla97Chr10	29702684	C	C	23|23	C	C	25|25	T
Cla97Chr10	29705538	C	C	33|33	C	C	25|25	T
Cla97Chr10	29706274	C	C	22|22	C	C	29|29	A
Cla97Chr10	29710794	T	T	21|21	T	T	24|24	C
Cla97Chr10	29712390	G	G	10|10	G	G	22|22	C
Cla97Chr10	29712772	T	T	11|11	T	T	19|19	C
Cla97Chr10	29713911	G	G	25|25	G	G	27|27	A
Cla97Chr10	29714236	G	G	23|23	G	G	25|25	C
Cla97Chr10	29715035	G	G	25|25	G	G	24|24	A
Cla97Chr10	29715935	G	G	18|18	G	G	19|19	A
Cla97Chr10	29720960	A	A	30|30	A	A	32|32	G
Cla97Chr10	29721039	T	T	24|24	T	T	25|25	C
Cla97Chr10	29724360	G	G	18|18	G	G	26|26	A
Cla97Chr10	29725056	T	T	20|20	T	T	28|28	C
Cla97Chr10	29725293	C	C	24|24	C	C	25|25	A
Cla97Chr10	29759694	C	C	18|18	C	C	21|21	T
Cla97Chr10	29762765	G	G	23|23	G	G	21|21	A
Cla97Chr10	29782306	A	A	20|20	A	A	28|28	G
Cla97Chr10	29790057	T	T	18|18	T	T	25|25	C
Cla97Chr10	29792075	A	A	27|27	A	A	28|28	G
Cla97Chr10	29819233	G	G	11|12	G	G	16|19	A

45NC_depth	DRDSBA	DRDSBA_dep	SBA	SBA_depth	Genic/Interg	Transcript.fe	Feature	Description
16|16	A	11|11	T	9|9	Intergenic			
12|12	A	14|14	T	8|8	Intergenic			
25|25	T	33|33	C	30|30	Cla97C10G199460		promoter	
15|15	A	10|10	G	7|7	Intergenic			
14|14	A	29|29	G	19|19	Intergenic			
34|34	G	33|33	A	27|27	Intergenic			
15|15	G	17|17	A	18|18	Cla97C10G19	Cla97C10G19	Intron	ARM repeat s
24|24	G	29|29	T	26|26	Cla97C10G19	Cla97C10G19	CDS,exon	ARM repeat s
24|24	A	26|26	G	16|16	Intergenic			
22|22	G	28|28	A	22|22	Intergenic			
18|18	G	21|21	C	15|15	Intergenic			
24|24	A	21|21	G	8|8	Intergenic			
22|22	T	30|30	C	24|24	Intergenic			
17|19	A	28|28	T	20|20	Intergenic			
24|24	T	35|35	C	41|41	Cla97C10G19	Cla97C10G19	CDS,exon	Protein of un
26|26	C	28|28	T	38|38	Cla97C10G19	Cla97C10G19	Intron	Protein of un
29|29	A	17|17	T	15|15	Intergenic			
31|31	C	24|24	T	25|25	Intergenic			
22|22	G	40|40	A	22|22	Intergenic			
25|25	T	28|28	A	30|31	Intergenic			
24|24	C	28|28	A	21|21	Intergenic			
33|33	C	26|26	A	31|31	Intergenic			
30|30	G	33|33	A	24|24	Cla97C10G19	Cla97C10G19	Intron	40S ribosoma
27|28	A	35|35	G	26|26	Cla97C10G19	Cla97C10G19	Intron	40S ribosoma
22|22	A	34|34	G	22|23	Cla97C10G19	Cla97C10G19	Intron	40S ribosoma
22|22	G	18|18	T	22|22	Cla97C10G199490		promoter	
12|12	C	12|12	T	7|7	Cla97C10G199490		promoter	
14|14	T	13|13	A	8|8	Cla97C10G199490		promoter	
26|26	G	25|25	A	17|17	Cla97C10G199490		promoter	
17|17	T	29|29	C	29|29	Intergenic			
29|29	G	26|26	A	25|25	Intergenic			
25|25	T	20|20	C	15|15	Intergenic			
19|19	G	24|24	T	10|10	Intergenic			
8|8	A	11|11	G	4|4	Intergenic			
7|7	A	10|10	N	0|2	Intergenic			
6|6	A	7|7	N	0|2	Intergenic			
7|7	C	14|14	A	8|8	Intergenic			
14|14	C	26|26	T	21|21	Intergenic			
10|10	T	38|38	C	15|15	Intergenic			
9|9	T	37|37	C	13|13	Intergenic			
15|15	C	31|31	A	15|15	Intergenic			
9|9	C	33|33	Y	1,8|9	Intergenic			
16|16	C	25|25	T	14|14	Intergenic			
17|17	T	43|43	A	8|8	Intergenic			
21|21	C	34|34	T	18|18	Intergenic			
25|25	C	25|25	T	33|33	Intergenic			
21|22	C	40|40	T	33|33	Intergenic			

17|17	A	47|47	G	24|25	Intergenic			
20|20	C	46|46	A	24|24	Intergenic			
22|23	C	26|27	T	30|30	Intergenic			
17|17	G	17|17	A	17|17	Intergenic			
21|21	T	28|28	A	18|20	Intergenic			
29|29	A	21|21	G	33|33	Intergenic			
19|19	T	30|30	A	28|28	Intergenic			
20|20	C	28|28	T	31|31	Intergenic			
22|22	T	28|28	C	31|31	Intergenic			
15|15	C	21|21	A	20|20	Cla97C10G199500		promoter	
19|19	T	25|25	C	19|19	Cla97C10G199500		promoter	
18|18	C	29|29	T	28|28	Cla97C10G19	Cla97C10G19	Intron	kinesin-4-like
28|28	T	45|45	C	38|38	Cla97C10G19	Cla97C10G19	Intron	kinesin-4-like
23|23	G	29|29	A	33|33	Cla97C10G19	Cla97C10G19	CDS,exon	kinesin-4-like
23|23	A	20|20	G	33|33	Intergenic			
24|24	A	41|41	T	25|25	Intergenic			
21|21	C	35|35	T	25|25	Intergenic			
22|22	T	14|14	C	16|16	Intergenic			
26|26	G	14|14	A	13|13	Cla97C10G199550		promoter	
30|30	G	25|25	A	16|16	Intergenic			
33|33	G	26|26	C	29|29	Intergenic			
20|20	A	36|36	T	30|30	Cla97C10G19	Cla97C10G19	CDS,exon	Arf GTPase a
32|32	G	25|25	A	22|22	Cla97C10G19	Cla97C10G19	Intron	Carboxypepti
21|21	A	20|20	T	16|16	Cla97C10G19	Cla97C10G19	Intron	Carboxypepti
31|31	A	38|38	G	17|17	Cla97C10G19	Cla97C10G19	Intron	regulation of
18|18	C	30|30	T	24|25	Cla97C10G19	Cla97C10G19	Intron	regulation of
18|18	G	34|34	A	29|29	Cla97C10G19	Cla97C10G19	Intron	regulation of
27|28	A	23|24	T	16|16	Cla97C10G19	Cla97C10G19	Intron	regulation of
19|19	A	25|26	T	26|26	Cla97C10G19	Cla97C10G19	Intron	regulation of
27|27	T	29|29	C	29|29	Cla97C10G19	Cla97C10G19	Intron	regulation of
34|34	C	39|39	T	26|26	Cla97C10G19	Cla97C10G19	Intron	regulation of
12|12	T	28|28	C	12|12	Cla97C10G19	Cla97C10G19	Intron	regulation of
25|25	T	20|20	C	26|26	Cla97C10G19	Cla97C10G19	Intron	regulation of
25|25	T	37|37	A	32|32	Cla97C10G19	Cla97C10G19	Intron	regulation of
18|18	A	47|47	T	34|34	Cla97C10G19	Cla97C10G19	Intron	regulation of
14|14	T	32|32	C	14|14	Intergenic			
24|24	C	43|43	A	24|24	Cla97C10G19	Cla97C10G19	CDS,exon	membrane st
31|31	C	38|38	A	39|39	Cla97C10G199630		promoter	
19|19	G	37|37	T	28|28	Cla97C10G19	Cla97C10G19	Intron	protein PLAN
15|15	A	20|20	T	12|12	Intergenic			
35|35	A	19|19	G	23|23	Intergenic			
26|26	G	21|21	A	20|20	Intergenic			
22|22	A	40|40	G	37|37	Intergenic			
21|21	G	20|20	A	20|20	Intergenic			
33|33	C	27|27	A	26|27	Intergenic			
19|19	T	11|11	C	7|7	Intergenic			
19|19	G	20|20	A	7|7	Intergenic			
22|22	T	19|19	C	18|18	Intergenic			
22|22	A	28|28	G	38|38	Intergenic			
16|16	A	20|20	C	20|20	Intergenic			

14|14	A	19|19	G	18|18	Intergenic			
29|29	A	19|19	G	25|25	Intergenic			
38|38	T	36|36	G	35|35	Intergenic			
20|20	C	32|32	T	19|19	Intergenic			
28|28	T	35|36	A	34|34	Intergenic			
27|27	A	29|29	T	21|21	Intergenic			
17|17	T	22|22	A	20|20	Intergenic			
7|7	G	12|12	A	7|7	Cla97C10G19	Cla97C10G19	Intron	14 kDa zinc-b
16|16	T	42|42	C	21|21	Cla97C10G199740		promoter	
11|11	A	7|7	T	4|4	Intergenic			
25|25	C	38|38	T	26|26	Intergenic			
3|3	C	9|9	T	3|3	Cla97C10G19	Cla97C10G19	Intron	MYB transcri
18|18	T	20|20	C	16|16	Intergenic			
36|36	G	39|39	A	29|29	Intergenic			
15|15	A	19|19	T	16|16	Cla97C10G199760		promoter	
27|27	C	43|43	T	28|28	Cla97C10G19	Cla97C10G19	Intron	Ribosome pr
27|27	T	35|35	G	26|26	Intergenic			
30|30	A	21|21	C	7|7	Cla97C10G199770		promoter	
22|23	T	41|41	C	33|33	Cla97C10G19	Cla97C10G19	Intron	Unknown pro
23|23	G	45|45	T	29|29	Cla97C10G19	Cla97C10G19	Intron	Unknown pro
16|16	C	33|33	T	23|23	Cla97C10G19	Cla97C10G19	Intron	Unknown pro
29|29	C	33|33	T	30|30	Cla97C10G19	Cla97C10G19	Intron	Unknown pro
21|21	C	39|39	A	27|27	Cla97C10G19	Cla97C10G19	Intron	Unknown pro
20|20	T	42|42	C	24|24	Cla97C10G19	Cla97C10G19	Intron	Unknown pro
21|21	G	33|33	C	27|27	Cla97C10G19	Cla97C10G19	Intron	Unknown pro
11|11	T	17|17	C	25|25	Cla97C10G19	Cla97C10G19	Intron	Unknown pro
21|21	G	23|23	A	25|25	Cla97C10G19	Cla97C10G19	Intron	Unknown pro
31|31	G	36|36	C	32|32	Cla97C10G19	Cla97C10G19	Intron	Unknown pro
35|35	G	29|29	A	38|38	Cla97C10G19	Cla97C10G19	Intron	Unknown pro
27|27	G	35|35	A	29|29	Cla97C10G19	Cla97C10G19	Intron	Unknown pro
35|35	A	21|21	G	39|40	Cla97C10G19	Cla97C10G19	Intron	Unknown pro
22|22	T	38|38	C	28|28	Cla97C10G19	Cla97C10G19	Intron	Unknown pro
20|20	G	30|31	A	32|32	Cla97C10G19	Cla97C10G19	Intron	Unknown pro
25|25	T	23|23	C	22|22	Cla97C10G19	Cla97C10G19	Intron	Unknown pro
25|25	C	34|34	A	26|26	Cla97C10G19	Cla97C10G19	Intron,promo	Unknown pro
15|15	C	12|12	T	9|9	Cla97C10G19	Cla97C10G19	Intron	Pleiotropic dr
18|18	G	25|25	A	16|16	Cla97C10G19	Cla97C10G19	Intron	Pleiotropic dr
30|30	A	22|22	G	22|22	Cla97C10G19	Cla97C10G19	Intron	Pleiotropic dr
24|24	T	34|34	C	16|16	Cla97C10G199810		promoter	
35|35	A	25|25	G	21|21	Intergenic			
21|21	G	13|13	A	10|10	Intergenic			

Interpro	GO	GO.desc	EC.number	Flanking_seq.(600bp)	Allele
				AAAAATGAAAAATTTCGGAAT	A/T
				AATATAAAGGGTCAAATTGGA	A/T
				TGGGTATGGTTTTGATTGGAC	T/C
				AATTGAGGGTTATAAGAATAT	A/G
				TTAATTTATATAAATTGAAGAT	A/G
				TTTATATAATGCATCACACATA	G/A
IPR016024 (Armadillo-type		uncharacteriz	NA	TTTGGGATTGACAATCTTACTA	G/A
IPR016024 (Armadillo-type		uncharacteriz	NA	TAGCCAGGTTTCTATAATGCTT	G/T
				CAAGTTTGAAATTTATGTGACC	A/G
				GATTCAAATTGATAAGTTTGAT	G/A
				TTAAGAATTATTTTGAAAAATC	G/C
				TACTATTGAAATGGGGAGAGA	A/G
				CATTCTCATCTTTCATTTGGTTC	T/C
				TAAATAGCTCATTGTAGCTACT	A/T
IPR005049 (Protein of unkn		probable glyc	NA	TATCAAACTCTCTTTTCCTTTTT	T/C
IPR005049 (Protein of unkn		probable glyc	NA	CAATCTGAGCTTTCAACGGAG	C/T
				ACACAAATACAAAATTTAGTTC	A/T
				AATAATTATACTACTTTAAATT	C/T
				CTAAGCACAAACTACAAAGGG	G/A
				AAGGAAATGTTTAGTCGGATA	T/A
				AAGCACAGCTAAATTGACATA	C/A
				TACATATAGATCATTAAGCAAT	C/A
IPR000851 (R	GO:0003723,	40s ribosoma	NA	GTGGGTTTGGCCAATAAATCC	G/A
IPR000851 (R	GO:0003723,	40s ribosoma	NA	CCGTGTGCTCTTGGAATGGAG	A/G
IPR000851 (R	GO:0003723,	40s ribosoma	NA	TAAATTTCTTATGATTAAGATT	A/G
				CTGGCATAATCTTCATAACCTC	G/T
				GACGGAGAGAGGAATTTATAT	C/T
				AGTGGCCCGGAGTTCCTATAA	T/A
				TAATAACTTACATTAACACTAA	G/A
				TTTAAAAAGTGGTAATTGTTGT	T/C
				CAAAATAAACCAATACGAGCA	G/A
				GGGTAGTCACTAGAAACCTTG	T/C
				GAGAATATGAAGTGTTTAAAA	G/T
				TTGGGTAAATCGGTCAAAGTG	A/G
				TCGGTCAAAGTGTTGGAGTCT	A/G
				AGTCTAAAGTTGGAATTCCATT	A/G
				GAGCAAAATGACAGAAAAACC	C/A
				AACTAAATTAAATAAACTTAAA	C/T
				ATGTCTCGTTAAAACCTTACCA	T/C
				GATCATGAGTGTAGAAAAGTT	T/C
				GCCAATATATAGCAGTTCCTCC	C/A
				AGATAAATATCCAGAATCTGC	T/C
				AATAATGGAGTATATGCTTAAT	C/T
				CCATATAAAATCTTTTTAAAAT	T/A
				TTGAATTATTTTGATATCCTTCT	C/T
				TCCAAATTTTCATACACAGTCA	C/T
				TTTTTCGAGGATTTTTATCTTTC	C/T

			ACGTTTAATAAATGATTCTGC	A/G
			TGCTAGACATAGATCTTCATT	C/A
			TCTCAAAATAAAAAAAAACAT	C/T
			TTAATGAATATTTTATTCTTTC	G/A
			TTAAGTTTGATCAAAATAAGT	T/A
			GTCCGAATGGAAAATAAAAA	A/G
			TTCAACAATAAATTATCCTCAT	T/A
			TTTTTTCTTTTCCAGCAATTGG	C/T
			CAATTGGAATTGAAATATCAA	T/C
			TTCTCTCTCTTTTGATGTCATT	C/A
			CTCTTTTGATGTCATTCTCCTT	T/C
IPR001715 (C	GO:0000166, kinesin-like p	NA	AAAGCAAGGTCGTTATGCTGA	C/T
IPR001715 (C	GO:0000166, kinesin-like p	NA	TGGTCCGAATGGTGCTACTAA	T/C
IPR001715 (C	GO:0000166, kinesin-like p	NA	ACTCTCAAATGTTTATCTCAGT	G/A
			TGGGATCCAAGGCTCTAATAT	A/G
			AGGAAACATGGAAGTTAGCA	A/T
			ACCAAGTCTAGCACAATGAG	C/T
			AACATTGACATGACATCACCG	T/C
			TTGTATGATTAAAGTGTGATG	G/A
			GGAGATCATAGGATACTTCCA	G/A
			ATAATTGAGTAGAGTGAGTTT	G/C
IPR001164 (A	GO:0005096, probable adp	NA	ACCTAGTACATCAGGAGTTTT	A/T
IPR001563 (P	GO:0004180, serine carbox	EC:3.4.16.0	AAAATGATGACATAGAATATT	G/A
IPR001563 (P	GO:0004180, serine carbox	EC:3.4.16.0	TAGAATTTTTTAGCTATATTTA	A/T
IPR006569 (CID domain), I	NA	NA	ATTTCCAGTATGCTTATAAAA	A/G
IPR006569 (CID domain), I	NA	NA	CTCAAGCATCACAGCCATTGA	C/T
IPR006569 (CID domain), I	NA	NA	TTGTTTAGAAACTAATAAAGC	G/A
IPR006569 (CID domain), I	NA	NA	CTAGAAGGGTATGGACATAA	A/T
IPR006569 (CID domain), I	NA	NA	TCCAAACCAAAAAGATGCAAC	A/T
IPR006569 (CID domain), I	NA	NA	AACTTGTGAAACAGAACGCAT	T/C
IPR006569 (CID domain), I	NA	NA	CGAGCCAATTCAAAACACTTA	C/T
IPR006569 (CID domain), I	NA	NA	CCGCGGATATTTTCTTTTTCTG	T/C
IPR006569 (CID domain), I	NA	NA	TCCCAAACAAAATTAACAAAC	T/C
IPR006569 (CID domain), I	NA	NA	GAAGGGATGATGATAGAAGT	T/A
IPR006569 (CID domain), I	NA	NA	TGGTGTAAATTGTCACCTCCT	A/T
			CATAATTCATATTAAGTTGAA	T/C
IPR001199 (C	GO:0016020, membrane st	NA	AATATGTAGACCTTATTATTA	C/A
			ATTCAGAAGCGGAGATCATTT	C/A
IPR006461 (Uncharacterise	protein plant	NA	AAATGGGTAGCTCATTGCAAC	G/T
			ATTTTTTAAAATAAATTTTAAA	A/T
			CTGGCCAGCTTGATTTTTTTAT	A/G
			GTTTTTAAGAAATCAAAATAG	G/A
			ATAAAGGTGGGATGACGAAA	A/G
			TTTTAAGTAAACAAACCATCA	G/A
			AGTATTGTTTTGAAATTATTG	C/A
			AAACATCAATTTATGATTAAA	T/C
			TTGACTTTTTTTTTTCTTTTTTG	G/A
			AAGAATCACTAAATTTGAAAT	T/C
			CATTTTTTATGACATTAGTATT	A/G
			CATCTCTATTCTATTTTATAAT	A/C

				ATTTTATAATAAAAGGAACAT	A/G
				TACATTCTTCATTACATTCTGT	A/G
				GATATGTGTAATTTGATATGT	T/G
				TAGGAACTCAAACATGATAAG	C/T
				TAATAGAACCAAGAACTATTC	T/A
				TTGGTTCTATAAGCAACCAAA	A/T
				GGAAAATAATCGTCATTTGGA	T/A
IPR001310 (H	GO:0003824	14 kda zinc-b	EC:3.6.2.1	TGTTTTTTATGAAGTCTTTGTG	G/A
				GCTTAAAACTGAGAGCCGAG	T/C
				TATAAAATGCAAGAGTTATAT	A/T
				TATCAGTCTTTCTCCAATGTAA	C/T
IPR009057 (H	GO:0003677,	myb family tr	NA	GTAATTAATTATCTGTCTCTAA	C/T
				TTATAGGGTAAGACTCTACTT	T/C
				TTTACCTTGTTACCAAACGTAT	G/A
				GTAAAATATTGTCGAAAGCTT	A/T
IPR004154 (Anticodon-bin		ribosome prc	NA	CATCATCGGTGAGATACTATA	C/T
				GTTGCTATCGTCACAATTTCA	T/G
				TCGTGACACAAGTGTTATCCA	A/C
tein		NA	NA	AGTTATTTTCAATGGACATGG	T/C
tein		NA	NA	CATTTGAGGACCTAATCGAAA	G/T
tein		NA	NA	ATGTACGGAGGAAGGAAGAT	C/T
tein		NA	NA	TACAAGGCGGAAGGGCAAGA	C/T
tein		NA	NA	GGACTACTGCATAGGATACAC	C/A
tein		NA	NA	TGGATATAAGATTTTAGTCAA	T/C
tein		NA	NA	GCTGTATAAAAGACAGCCAAC	G/C
tein		NA	NA	CTTTTTGTTAAGTCGAGTCGC	T/C
tein		NA	NA	AGGTTTATTGCTACGTATGTA	G/A
tein		NA	NA	CATATTTATATTTTACCCGAAA	G/C
tein		NA	NA	CTATCTTTAACATGGAGGTTT	G/A
tein		NA	NA	GAAGTCATTTTCCATTGACAT	G/A
tein		NA	NA	AGTAAGATAGGTTACAAGAA	A/G
tein		NA	NA	TTTGGATAAAGGATTAGCAAA	T/C
tein		NA	NA	GAAGCTCCTTATTGTTGTATA	G/A
tein		NA	NA	CCATCAGTTTGACCCATTGAC	T/C
tein		NA	NA	AACTACTGTGGGGGTGGGGT	C/A
IPR003439 (A	GO:0000166,	pleiotropic d	EC:3.6.1.3	TTATATTATTATACATATTTTA	C/T
IPR003439 (A	GO:0000166,	pleiotropic d	EC:3.6.1.3	AACTGTCCAATCCATTTGTTAT	G/A
IPR003439 (A	GO:0000166,	pleiotropic d	EC:3.6.1.3	CTAATTTATTTATAAATATAGC	A/G
				ATATTGTAGATTTGTGGAAAT	T/C
				CTACTATTTATGGGTGTTTTAG	A/G
				GACCAAGGTCGTTTAGTCAAT	G/A
