# Supplementary material for: Whole-Genome Resequencing of Near-Isogenic Lines Reveals a Genomic Region Associated with High Trans-Lycopene Contents in Watermelon
Source: Plants (Basel). 2021 Dec 21;11(1):8. doi: 10.3390/plants11010008 (PMC8747524; doi:10.3390/plants11010008)
Supplement: Supplementary file 1 [file plants-11-00008-s001.zip › Table S3.pdf]

**Table S4.** Cleaved amplified polymorphic sequence (CAPS) markers developed from the common introgression regions in two near-isogenic lines.

| Marker ID | Position | | Gene ID | SNP (D/R)[a] | Primer sequence (5′-3′) | Enzyme | Size (bp) (D/R)[a] |
|---|---|---|---|---|---|---|---|
| | Chr. | bp | | | | | |
| CIR6-M1 | 6 | 24514564 | Cla97C06G122150 | C/T | F:AGGGAGATTGTGAAGTAGAGAGCTT<br>R:GGAATTTCATATTTTCTTCCGAACT | *Mse*I | 345, 88/295, 50, 88 |
| CIR6-M2 | 6 | 24590478 | Cla97C06G122230 | G/A | F:AGTGAAAACAGGTGAGAATGCTAAT<br>R:CATTTCATCTTGAACTTCATTTTCC | *Fok*I | 184, 30/214 |
| CIR6-M3 | 6 | 24620757 | Cla97C06G122260 | T/C | F:CTGAACCTTATCAATAGCTTTCCAA<br>R:CTTGAGAATGCTGAGATTATCCATT | *Hpy188*III | 140, 123/263 |
| CIR6-M4 | 6 | 24687928 | Cla97C06G122350 | A/G | F:CTGAAGTCTTTAGGCACTGGACTAC<br>R:TTAAACAAATGTCTTTGATGTCAGG | *Mse*I | 55, 162, 3/217, 3 |
| CIR6-M5 | 6 | 24751036 | Cla97C06G122390 | T/C | F:ACAGTGACTGCTATATCCTTCCAAC<br>R:CTAGATGGGAATCTCAAGGTATTCA | *Rsa*I | 87, 143/230 |
| CIR6-M6 | 6 | 24810309 | Cla97C06G122460 | T/C | F:TCAGAACTGTAATGAACGAACCTAA<br>R:TCGAAAGAATTTATGAGAACAGAGG | *Dde*I | 22, 37, 123, 88/22, 37, 211 |
| CIR6-M7 | 6 | 24838861 | Cla97C06G122510 | A/G | F:AGAACTCAACTTCATCATCTCCAAC<br>R:TGAACGTAGATATTATGGGTTCGAT | *Taq*I | 48, 103, 95, 23/48, 198, 23 |
| CIR6-M8 | 6 | 24851621 | Cla97C06G122540 | G/A | F:GATCAATATCGAACCCACAGTAATC<br>R:GAGCAAATCATGTTCTCAATCTTTT | *Alu*I | 89, 140/229 |
| CIR9-M1 | 9 | 1647313 | Cla97C09G163980 | G/A | F:CCTTCACAGTCTTCAAAGTAACACA<br>R:CTCTTCGGTAATGATTTTGAGGATA | *Hha*I | 96, 181/277 |
| CIR9-M2 | 9 | 1771791 | Cla97C09G164160 | C/A | F:AAACCCAATACAATTTTTCATCAGA<br>R:ATAACTGGAGTGTGAAGGCAATTAG | *Mnl*I | 108, 46, 45/154, 45 |
| CIR9-M3 | 9 | 1863987 | Cla97C09G164320 | T/G | F:CTAGTTTCTGCCTGTTCAGAGATTC<br>R:TGATAGCAATCCTAAGATTTTCCTG | *MluC*I | 102, 43, 36, 61, 38/145, 36, 61, 38 |
| CIR9-M4 | 9 | 1865138 | Cla97C09G164320 | T/C | F:GTGAAATATTGAGGCTTAGGAGTGA<br>R:GTAACAGGATAACCAGATGTTGGAG | *MluC*I | 143, 88/231 |
| CIR9-M5 | 9 | 1919043 | Cla97C09G164360 | T/G | F:GCGACTCAAAAAGATGGAGATAATA<br>R:CTTACCACCACTTACACATTCATCA | *Apo*I | 87, 186/273 |
| CIR9-M6 | 9 | 2135592 | Cla97C09G164610 | G/T | F:GTGCAGCTGTGCTATACTATTCTGA<br>R:CATTCCCATACAGATCTCACTTTCT | *Bcl*I, | 160, 82/242 |
| CIR9-M7 | 9 | 2263785 | Cla97C09G164820 | G/A | F:GCTGACATGATATCTGGTGATGTAG<br>R:GTGATAACCTTGTCATCCTTGGTAT | *Aci*I | 175, 97/272 |
| CIR9-M8 | 9 | 3363590 | Cla97C09G166230 | G/A | F:TGATATGTCATCTTCAAAACAACCA<br>R:GAAGAAACAAAGAATTGCCAGATAG | *Dde*I | 164, 212/285 |
| CIR9-M9 | 9 | 3714121 | Cla97C09G166750 | C/A | F:CAACAATTTTTAGAAACCAATGGAG<br>R:TTTGGCTAAGAGGAAGGTTTTACTT | *Aci*I | 142, 103/245 |
| CIR9-M10 | 9 | 3759192 | Cla97C09G166820 | T/G | F:CCATTCTTCCCTCTTTTAAAACCT<br>R:CCTTAGTGTAGAAATCAACGGAATC | *Taq*I | 63, 206/63, 60, 146 |
| CIR9-M11 | 9 | 3834373 | Cla97C09G166910 | T/C | F:GATCATGGTTCATTTCCTCATCTAC<br>R:TTATTATCACGGAGAAAAGTTCTGG | *Hinf*I | 189/78, 111 |
| CIR9-M12 | 9 | 3876768 | Cla97C09G166950 | T/C | F:TGCGTAGCTACCAAAATTACTAAATG<br>R:ATTTGTTGCACCAAGGATAAGAAT | *Hinc*II | 285/150, 135 |
| CIR9-M13 | 9 | 3905300 | Cla97C09G166980 | C/T | F:TGAAATTCACCCTGAAGTAAAAGAA<br>R:AGAGTAAAAATTGCTGTAGGTGCTG | *Taq*I | 117, 89, 58/206, 58 |
| CIR10-M1 | 10 | 29440068 | Cla97C10G199470 | G/T | F:AGAGGACTGCAAGTTACAAGCTGTT<br>R:GAAGTTTCCTCTGACAGCTGATTTC | *Hinf*I | 350/131, 219 |
| CIR10-M2 | 10 | 29447001 | Cla97C10G199480 | T/C | F:AGTTGATGAATTTGTAGCAGGTGGT<br>R:TACCCAAATCCTGGACTACTGTCTC | *Hae*III | 350/148, 202 |
| CIR10-M3 | 10 | 29476855 | Cla97C10G199500 | G/A | F:CTCATTGCATGGTAATCTTCATTTGGT<br>R:GGGATTAAGCTGTACAAACATGACC | *Hpy*188I | 61, 137, 170/61, 307 |
| CIR10-M4 | 10 | 29548144 | Cla97C10G199570 | A/T | F:TAGAGACGGATCATGAGCAATGTAA<br>R:AAGATCAACCTCAACAAAAGTCACC | *Mbo*II | 72, 64, 55, 147/71, 64, 202 |
| CIR10-M5 | 10 | 29676339 | Cla97C10G199740 | T/C | F: CGGAACCTCTATAATTTCAACGCAAA<br>R: CATGGAAAGATGTGAGCAATGGTATT | *Aci*I | 262/72, 190 |

[a]D, donor parent (DRD); R, recurrent parents (SBA and 45NC)
