# Supplementary material for: Whole-Genome Resequencing of Near-Isogenic Lines Reveals a Genomic Region Associated with High Trans-Lycopene Contents in Watermelon
Source: Plants (Basel). 2021 Dec 21;11(1):8. doi: 10.3390/plants11010008 (PMC8747524; doi:10.3390/plants11010008)
Supplement: Supplementary file 1 [file plants-11-00008-s001.zip › Table S4.pdf]

Table S3. List of 16 genes involved in the lycopene biosynthesis pathway

Gene	Annotation	Description	Chromosome number	Position (bp)
GGPP	Cla97C02G039100	Geranylgeranyl pyrophosphate synthase	2	26782617..26783708
PSY1	Cla97C01G008760	Phytoene Synthase	1	9448426..9451311
PSY1	Cla97C01G024630	9-cis-epoxycarotenoid dioxygenase	1	35670071..35671885
PDS	Cla97C07G137500	Phytoene synthase 3	7	25039090..25042269
PDS	Cla97C07G142100	Phytoene desaturase	7	29622146..29638496
PDS	Cla97C08G151960	3-hydroxy-3-methylglutaryl coenzyme A reductase	8	20348534..20351635
PDS	Cla97C08G156390	1-deoxy-D-xylulose-5-phosphate synthase 3	8	24212801..24217275
ZDS	Cla97C06G118930	Zeta-carotene desaturase	6	14092257..14103221
CRTISO	Cla97C10G200950	Carotenoid isomerase	10	31017567..31022669
LCYB	Cla97C04G070940	Lycopene beta-cyclase	4	15442312..15443826
LCYE	Cla97C11G208040	Lycopene epsilon cyclase	11	1758127..1763676
NCED	Cla97C07G137260	9-cis-epoxycarotenoid dioxygenase 1	7	24786951..24788687
CHYb	Cla97C01G002480	Beta-carotene hydroxylase	1	2231086..2233928
CHYb	Cla97C01G010890	Acetyl-CoA C-acetyltransferase	1	17211471..17218294
ZEP	Cla97C02G038200	Zeaxanthin epoxidase, chloroplastic	2	25484173..25490762
VDE	Cla97C11G216330	Violaxanthin de-epoxidase	11	17320033..17327558
