# Supplementary figures and images for: Whole-Genome Resequencing of Near-Isogenic Lines Reveals a Genomic Region Associated with High Trans-Lycopene Contents in Watermelon
Source: Plants (Basel). 2021 Dec 21;11(1):8. doi: 10.3390/plants11010008 (PMC8747524; doi:10.3390/plants11010008)

Figure S2: Introgressed regions in DRDSBA (A) and DRD45NC (B) detected based on the frequencies of SNPs

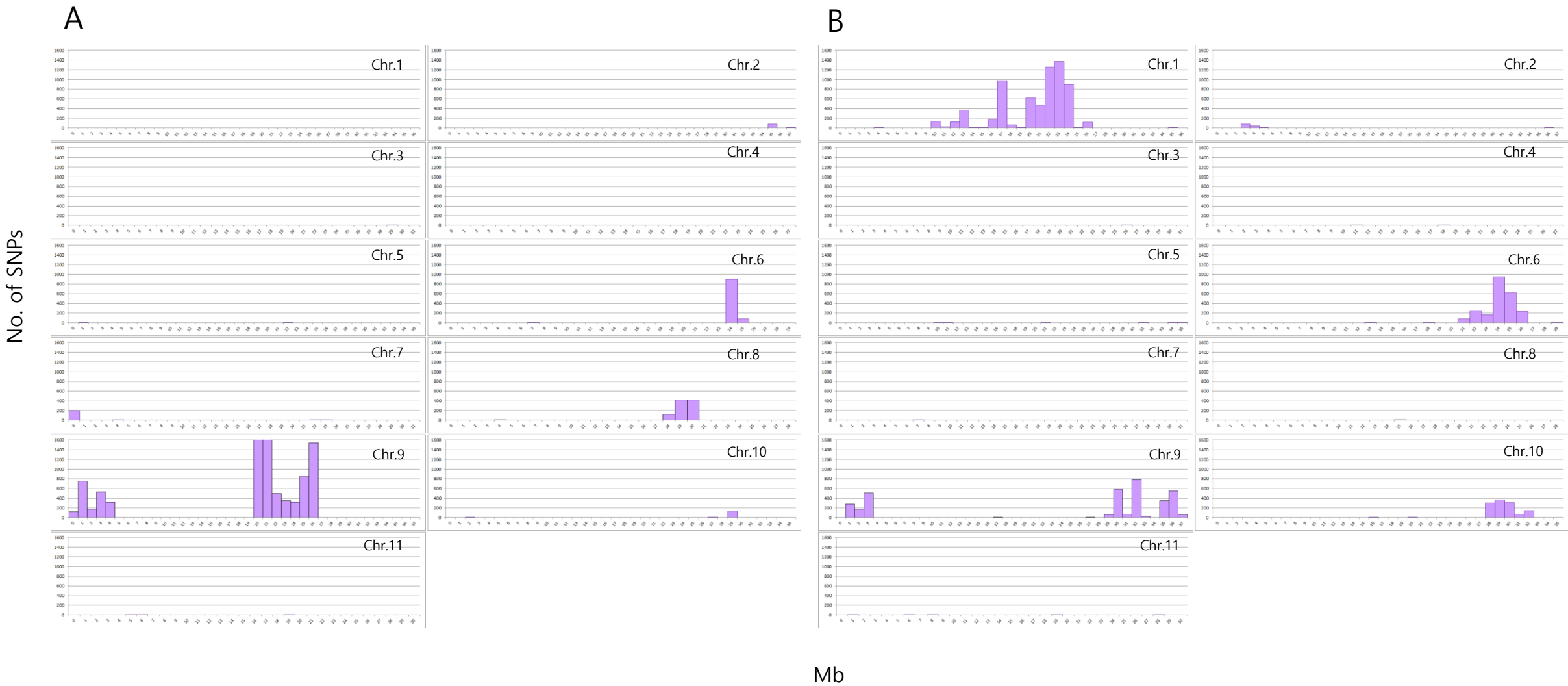

Supplement: Supplementary file 1 [file plants-11-00008-s001.zip › Figure S2.pdf]
